# Supplementary material for: ‘Immunity Passports’ for SARS-CoV-2: an online experimental study of the impact of antibody test terminology on perceived risk and behaviour
Source: BMJ Open. 2020 Aug 30;10(8):e040448. doi: 10.1136/bmjopen-2020-040448 (PMC7462240; doi:10.1136/bmjopen-2020-040448)
Supplement: Supplementary data [file bmjopen-2020-040448supp001.pdf]

Supplementary Materials

S1 Interventions

Intervention A

Immunity Passport

Scientists are developing tests to see who has already had coronavirus.

No test is 100% effective.

This means that those who test 'positive' would have:

- Lower risk of catching coronavirus in the future - and therefore also
- Lower risk of passing it on to others

Those who test 'positive' would get an immunity passport.

They could return to work early.

Intervention B

Immunity Certificate

Scientists are developing tests to see who has already had coronavirus.

No test is 100% effective.

This means that those who test 'positive' would have:

- Lower risk of catching coronavirus in the future - and therefore also
- Lower risk of passing it on to others

Those who test 'positive' would get an immunity certificate.

They could return to work early.

Intervention C

Immunity Test

Scientists are developing tests to see who has already had coronavirus.

No test is 100% effective.

This means that those who test 'positive' would have:

- Lower risk of catching coronavirus in the future - and therefore also
- Lower risk of passing it on to others

Those who test 'positive' would get a result showing immunity.

They could return to work early.

Intervention D

Antibody Passport

Scientists are developing tests to see who has already had coronavirus.

No test is 100% effective.

This means that those who test 'positive' would have:

- Lower risk of catching coronavirus in the future - and therefore also
- Lower risk of passing it on to others

Those who test 'positive' would get an antibody passport.

They could return to work early.

Intervention E

Antibody Certificate

Scientists are developing tests to see who has already had coronavirus.

No test is 100% effective.

This means that those who test 'positive' would have:

- Lower risk of catching coronavirus in the future - and therefore also
- Lower risk of passing it on to others

Those who test 'positive' would get an antibody certificate.

They could return to work early.

Intervention F

Antibody Test

Scientists are developing tests to see who has already had coronavirus.

No test is 100% effective.

This means that those who test 'positive' would have:

- Lower risk of catching coronavirus in the future - and therefore also
- Lower risk of passing it on to others

Those who test 'positive' would get a result showing antibodies.

They could return to work early.

S2. Measures

Primary outcome

I Imagine you were given *[an immunity passport/an immunity certificate/a result showing immunity/an antibody passport/an antibody certificate/a result showing antibodies]*

which one of the following statements is true*: [order of response options to be randomised]*

1. I have no risk of catching coronavirus in the future
2. I have a lower risk of catching coronavirus in the future
3. I have an average risk of catching coronavirus in the future
4. I have a higher risk of catching coronavirus in the future

Secondary outcomes

II Imagine you were given *[an immunity passport/an immunity certificate/a result showing immunity/an antibody passport/an antibody certificate/a result showing antibodies]*

how likely is it that you will get coronavirus at some point in the future?

Please answer on a scale from 0 % to 100% where 0% means no chance and 100% means certain.

0% --100%

III Imagine that you were given *[an immunity passport/an immunity certificate/a result showing immunity/an antibody passport/an antibody certificate/a result showing antibodies]*

would you wash your hands with soap and water or sanitiser:

1. Much more than now
2. More than now
3. Same as now
4. Less than now
5. Much less than now

IV Imagine you were given *[an immunity passport/an immunity certificate/a result showing immunity/an antibody passport/an antibody certificate/a result showing antibodies]*

would you avoid physical contact with others outside of your home:

1. Much more than now
2. More than now
3. Same as now
4. Less than now
5. Much less than now

V If you were offered a test today by the NHS to check whether you have ever had coronavirus, would you have it?

1. Yes, definitely
2. Yes, probably
3. No, probably not
4. No, definitely not

Supplementary Table 1 – Logistic regression analysis examining impact of Test type and Result type on protective behaviours

	Intend to wash hands less			Intend to avoid contact less		
	% responding less/much less in each sub-group % (95% CI)	Mutually adjusted (n=1204)	Adjusted for demographics[1] (n=1179)	% responding less/much less in each sub-group % (95% CI)	Mutually adjusted (n=1204)	Adjusted for demographics[1] (n=1179)
Test Type						
Antibody (n=603)	4.1 (2.7-6.1)	Ref	Ref	17.2 (104)	Ref	Ref
Immunity (n=601)	5.7 (4.0-7.8)	1.38 (0.43-4.45)	1.62 (0.46-5.73)	22.1 (133)	1.40 (0.82-2.39)	1.46 (0.85-2.50)
Result Type						
Test (n=354)	3.4 (1.8-5.9)	Ref	Ref	19.2 (68)	Ref	Ref
Certificate (n=444)	6.8 (4.6-9.5)	2.07 (0.72-6.00)	2.47 (0.77-7.96)	21.6 (96)	1.16 (0.68-1.96)	1.14 (0.67-1.96)
Passport (n=406)	4.2 (2.5-6.6)	1.29 (0.41-4.01)	1.79 (0.52-6.16)	18.0 (73)	0.99 (0.58-1.69)	1.02 (0.59-1.76)
Test by Result interaction						
Certificate by Immunity result		0.98 (0.25-3.95)	0.80 (0.18-3.56)		1.00 (0.49-2.01)	0.94 (0.46-1.93)
Passport by Immunity result		0.96 (0.21-4.40)	0.58 (0.11-2.98)		0.89 (0.43-1.87)	0.84 (0.40-1.78)

[1]Fully adjusted model includes age (with quadratic term), gender, education level and region (coded as per Table 1)

Supplementary Table 2 – Logistic regression examining impact of Test and Result type on intention to have the test

		Do not intend to have the test	
	Proportion who would not have test in each sub-group % (95% CI)	Mutually adjusted (n=1204)	Adjusted for demographics[1] (n=1179)
Test Type			
Antibody (n=603)	13.1 (10.5-16.1)	Ref	Ref
Immunity (n=601)	16.5 (13.6-19.7)	1.02 (0.57-1.81)	0.97 (0.54-1.74)
Result Type			
Test (n=354)	15.5 (11.9-19.7)	Ref	Ref
Certificate (n=444)	14.9 (11.7-18.5)	0.71 (0.39-1.28)	0.63 (0.34-1.15)
Passport (n=406)	14.0 (10.8-17.8)	0.80 (0.54-1.42)	0.82 (0.46-1.47)
Test by Result interaction			
Certificate by Immunity result		1.65 (0.75-3.63)	1.91 (0.84-4.31)
Passport by Immunity result		1.23 (0.55-2.75)	1.22 (0.54-2.78)

[1]Fully adjusted model includes age (with quadratic term), gender, education level and region (coded as per Table 1)

Supplementary Table 3 – Logistic regression analysis examining impact of perceived risk on anticipated behaviour

	Intend to wash hands less			Intend to avoid contact less		
	% responding less/much less in each sub-group % (95% CI)	Unadjusted (n=1204)	Adjusted for demographics[1] (n=1179)	% responding less/much less in each sub-group % (95% CI)	Unadjusted (n=1204)	Adjusted for demographics[1] (n=1179)
Perceived meaning of result						
Some residual risk (n=1030)	4.0 (2.8-5.3)	Ref	Ref	18.8 (16.5-21.4)	Ref	Ref
No residual risk of future infection (n=175)	10.3 (6.3-15.9)	**2.78 (1.56-4.97)**	**2.32 (1.25-4.28)**	24.7 (18.5-31.8)	1.41 (0.97-2.06)	1.37 (0.93-2.03)

[1]Fully adjusted model includes age (with quadratic term), gender, education level and region (coded as per Table 1)
