# Supplementary material for: ‘Immunity Passports’ for SARS-CoV-2: an online experimental study of the impact of antibody test terminology on perceived risk and behaviour
Source: BMJ Open. 2020 Aug 30;10(8):e040448. doi: 10.1136/bmjopen-2020-040448 (PMC7462240; doi:10.1136/bmjopen-2020-040448)
Supplement: Author's manuscript [file bmjopen-2020-040448.draft_revisions.pdf]

BMJ Open is committed to open peer review. As part of this commitment we make the peer review history of every article we publish publicly available.

When an article is published we post the peer reviewers' comments and the authors' responses online. We also post the versions of the paper that were used during peer review. These are the versions that the peer review comments apply to.

The versions of the paper that follow are the versions that were submitted during the peer review process. They are not the versions of record or the final published versions. They should not be cited or distributed as the published version of this manuscript.

BMJ Open is an open access journal and the full, final, typeset and author-corrected version of record of the manuscript is available on our site with no access controls, subscription charges or pay-per-view fees (http://bmjopen.bmj.com).

If you have any questions on BMJ Open's open peer review process please email info.bmjopen@bmj.com

BMJ Open

"Immunity Passports" for SARS-CoV-2: an online experimental study of the impact of antibody test terminology on perceived risk and behaviour

Journal:	*BMJ Open*
Manuscript ID	bmjopen-2020-040448
Article Type:	Original research
Date Submitted by the Author:	13-May-2020
Complete List of Authors:	Waller, Jo; UCL, Epidemiology and Public Health<br>Rubin, GJ; King\'s College London School of Medical Education<br>Potts, Henry; UCL, Centre for Health Informatics and Multiprofessional Education<br>Mottershaw, Abigail; Behavioural Insights Team<br>Marteau, Theresa; University of Cambridge, Behaviour and Health Research Unit
Keywords:	Public health < INFECTIOUS DISEASES, PUBLIC HEALTH, IMMUNOLOGY

SCHOLARONE™
Manuscripts

I, the Submitting Author has the right to grant and does grant on behalf of all authors of the Work (as defined in the below author licence), an exclusive licence and/or a non-exclusive licence for contributions from authors who are: i) UK Crown employees; ii) where BMJ has agreed a CC-BY licence shall apply, and/or iii) in accordance with the terms applicable for US Federal Government officers or employees acting as part of their official duties; on a worldwide, perpetual, irrevocable, royalty-free basis to BMJ Publishing Group Ltd ("BMJ") its licensees and where the relevant Journal is co-owned by BMJ to the co-owners of the Journal, to publish the Work in this journal and any other BMJ products and to exploit all rights, as set out in our licence.

The Submitting Author accepts and understands that any supply made under these terms is made by BMJ to the Submitting Author unless you are acting as an employee on behalf of your employer or a postgraduate student of an affiliated institution which is paying any applicable article publishing charge ("APC") for Open Access articles. Where the Submitting Author wishes to make the Work available on an Open Access basis (and intends to pay the relevant APC), the terms of reuse of such Open Access shall be governed by a Creative Commons licence – details of these licences and which Creative Commons licence will apply to this Work are set out in our licence referred to above.

Other than as permitted in any relevant BMJ Author's Self Archiving Policies, I confirm this Work has not been accepted for publication elsewhere, is not being considered for publication elsewhere and does not duplicate material already published. I confirm all authors consent to publication of this Work and authorise the granting of this licence.

For peer review only

"Immunity Passports" for SARS-CoV-2: an online experimental study of the impact of antibody test terminology on perceived risk and behaviour

Jo Waller PhD[1], G. James Rubin PhD[2], Henry W. W. Potts PhD[3], Abi Mottershaw[4], Theresa M Marteau PhD[5] *

[1] Jo Waller, Reader in Cancer Behavioural Science, School of Cancer and Pharmaceutical Sciences, King's College London, Guy's Hospital, Great Maze Pond, London, SE1 9RT.

[2] G. James Rubin, Reader in the Psychology of Emerging Health Risks. Department of Psychological Medicine, King's College London, Weston Education Centre, Cutcombe Road, London, SE5 9RJ.

[3] Henry W. W. Potts, Associate Professor, Institute of Health Informatics, University College London, 222 Euston Road London, NW1 2DA.

[4]Abigail L. Mottershaw, Online Experiments Lead. Behavioural Insights Team, 4 Matthew Parker Street, London, SW1H 9NP.

[*5] Theresa M Marteau, Director of Research, Behaviour and Health Research Unit, University of Cambridge, Institute of Public Health, Forvie Site, Robinson Way, Cambridge, CB2 0SR.

* corresponding author: tm388@cam.ac.uk

For peer review only

Objective: To assess the impact of describing an antibody-positive test result using the terms Immunity and Passport or Certificate, alone or in combination, on perceived risk of becoming infected with SARS-CoV-2 and intention to continue protective behaviours.

Design: 2 × 3 experimental design.

Setting: Online with data collected between 28th April and 1st May 2020.

Participants: 1,204 adults registered with a UK research panel.

Intervention: Participants were randomised to receive one of six descriptions of an antibody test and results showing SARS-CoV-2 antibodies, differing in the terms used to describe the type of test (Immunity *vs* Antibody) and the test result (Passport *vs* Certificate *vs* Test).

Main outcome measures: The primary outcome was the proportion of participants perceiving no risk of becoming infected with SARS-CoV-2 given an antibody positive test result. Other outcomes include intended changes to frequency of handwashing and physical distancing.

Results: When using the term Immunity (vs Antibody), 19.1% of participants [95% CI: 16.1 to 22.5] (vs 9.8% [95% CI: 7.5 to 12.4]) perceived no risk of catching coronavirus at some point in the future given an antibody-positive test result (AOR: 2.91 [95% CI: 1.52 to 5.55]). Using the terms Passport or Certificate – as opposed to Test – had no significant effect (AOR: 1.24 [95% CI: 0.62 to 2.48] and AOR: 0.96 [95% CI: 0.47 to 1.99] respectively). There was no significant interaction between the effects of the test and result terminology. Across groups, perceiving no risk of infection was associated with an intention to wash hands less frequently (AOR: 2.32 [95% CI: 1.25 to 4.28]) but there was no significant association with intended avoidance of physical contact with others outside of the home (AOR: 1.37 [95% CI: 0.93-2.03]).

Conclusions: Using the term Immunity (vs Antibody) to describe antibody tests for SARS-CoV-2 increases the proportion of people believing that an antibody-positive result means they have no risk of catching coronavirus in the future, a perception that may be associated with less frequent handwashing. The way antibody testing is

For peer review only

described may have implications for the likely impact of testing on transmission rates.

Key words: SARS-CoV-2; COVID-19; antibody testing; immunity; passport; certificate; risk perception; protective behaviours

Study registration: Open Science Framework: https://osf.io/tjwz8/files/

For peer review only

Strengths and limitations of this study

- This study provides the first experimental evidence for the potentially adverse impact on risk perceptions and protective behaviours of using the term Immunity – as opposed to Antibody - to describe SARS-CoV-2 antibody tests and their results.
- It provides timely evidence to inform policy and research to mitigate these effects to realise the potential benefits of such tests.
- Participants were responding to a hypothetical test and asked to imagine that they had received a test result that had detected antibodies.
- Protective behaviours of handwashing and physical distancing were measured using single items assessing behavioural intentions.

For peer review only - http://bmjopen.bmj.com/site/about/guidelines.xhtml

Introduction

At the height of the first wave of the COVID-19 pandemic, about a third of the world's population is estimated to have been in lockdown, with all but essential workers largely confined to home (1).

Without an effective treatment or vaccine, testing for infection combined with contact tracing and isolation will be central to effective strategies to ease populations out of lockdown while keeping the basic reproduction number (R_0) below one (2).

Testing for antibodies to SARS-CoV-2 is a possible complement to testing for active infection to identify those who have developed antibodies to the virus and so may be able to return to work and other activities without significantly increasing transmission rates (3). These tests have been variously described in the media as Immunity Passports (4,5), Immunity Certificates (6,7) Immunity Cards (8) and Release Certificates (9). Unfortunately, the use of these terms implies a certainty unmatched by current evidence about antibody tests (10).

Uncertainties inherent in tests for antibodies to SARS-CoV-2 include the extent and duration of immunity conferred (11). They also include the uncertainties inherent in any test regarding the proportion of those who would be correctly identified. This depends upon the test performance – its sensitivity and specificity – as well as the population prevalence of the tested condition (12). Given these uncertainties, those who receive a test result indicating the presence of antibodies will have a residual risk of becoming infected by SARS-CoV-2 in the future.

Understanding that there is this residual risk – albeit one that is difficult to quantify at present – will be important to minimise transmission that could arise from those receiving "antibody positive" test results. If people testing positive perceive that they have no risk of becoming infected by the virus, they may ignore any future symptoms of infection and facilitate transmission if they fail to self-isolate appropriately. Such a perception may also over-generalise to a belief that they are unable to transmit infection through contact with contaminated surfaces. Regardless of antibody status, all individuals can indirectly transmit the virus between surfaces by touch. Hand washing or sanitising therefore need to remain frequent.

Evidence from other testing programmes suggests that interpreting a low risk result to mean no risk can be reduced by verbal and numerical expressions of residual risk

For peer review only

when presenting test results (13,14). But even before testing programmes are in place, the terms commonly used to describe these tests – Immunity Passport or Certificates – may inadvertently be fuelling a misplaced sense of certainty about their results. It is unknown whether describing these tests as being for immunity – as opposed to antibodies – or their results as passports or certificates increases misunderstanding of the residual risk inherent in an antibody-positive test result and thereby reducing adherence to protective behaviours and increasing risk of transmission (15).

This study was designed to test two hypotheses: describing a test indicating the presence of antibodies using the term Immunity (vs Antibody), and describing test results as Passports or Certificates (vs Test), increases the likelihood that those with this test result erroneously perceive they have no risk of becoming infected in the future with coronavirus.

Methods

Ethical approval for this study was granted by the King's College London Research Ethics Committee (reference: MRA-19/20-18685).

The protocol was preregistered on the Open Science Framework https://osf.io/tjwz8/ *Study 2*

The statistical analysis plan was pre-specified and uploaded to the Open Science Framework prior to receipt of the data https://osf.io/tjwz8/ *Study 2*

An initial study with similar methods was conducted https://osf.io/tjwz8/ *Study 1* but, due to an error, the intervention was not correctly programmed. This study is therefore not reported.

Design

The study was an online experiment using a 2 × 3 factorial design, with participants randomised, with an equal allocation ratio, to one of six groups varying in the description of an antibody test and a result showing the presence of antibodies. These descriptions differed only in the term used for what was being tested

For peer review only - http://bmjopen.bmj.com/site/about/guidelines.xhtml

(Immunity *vs* Antibody) and the term used for the test result (Passport *vs* Certificate *vs* Test).

Participants

A quota sample of 1,204 adults was recruited via Predictiv, the Behavioural Insights Team's online experimentation platform (https://www.bi.team/bi-ventures/predictiv/) comprising 500,000 adults in the UK. Quotas were based on age, gender and UK region to achieve a sample broadly representative of the UK population. 1373 clicked on the link to enter the study of whom 1214 subsequently completed the study. Ten were excluded for failing to meet quality checks. Participants were reimbursed in points (equivalent to £1) which could be redeemed in cash, gift vouchers or charitable donations. Participants did not know the topic of the study prior to participation.

Patient and public involvement

Due to the rapid nature of this research, the public was not involved in the development of the study.

Power

The sample size was chosen pragmatically without reference to a specific power calculation. We are fitting a full model with an interaction. Conservatively, we then had an 80% chance of detecting, at a 5% significance level, an increase in the primary outcome measure from 50% in a baseline group to 64% in another group.

Randomisation

Participants were randomised to groups by random number generation. A random number between 1 and 6 was generated for every participant upon entry to the study to determine which description they saw, with each of the six numbers corresponding to one of the six description. As this is based on true randomness, the number of participants within each group can vary due to chance.

Intervention

The intervention comprised a description of antibody testing and test results indicating the presence of antibodies [see Box 1 for one example and S1 for wording of all six descriptions]. These differed across six groups in test name of results indicating the presence of antibodies. All descriptions included the information that the result would mean a lower risk of future infection and transmission, and that people with this result could return to work earlier.

For peer review only - http://bmjopen.bmj.com/site/about/guidelines.xhtml

Box Immunity Passport: one of the six descriptions of an antibody-positive test result

Immunity Passport

Scientists are developing tests to see who has already had coronavirus.

No test is 100% effective.

This means that those who test 'positive' would have:

- Lower risk of catching coronavirus in the future, and therefore also
- Lower risk of passing it on to others

Those who test 'positive' would get an immunity passport.

They could return to work early.

Outcomes measures

Wording of the items used for each measure is shown in Supplementary Materials (S2).

Primary outcome

Proportion of participants perceiving an antibody-positive test result to mean no risk of catching coronavirus in the future, assessed in response to a question with four response options.

Secondary outcomes

Perceived likelihood of catching coronavirus in the future, assessed on a visual analogue scale from 0% to 100%.

Intention to engage in handwashing less or more frequently than now, given an antibody-positive test result: assessed in response to a question with five response options.

For peer review only - http://bmjopen.bmj.com/site/about/guidelines.xhtml

Intention to avoid physical contact with others outside the home more or less frequently than now, given an antibody-positive test result: assessed in response to a question with five response options.

Interest in undergoing the test if offered today: assessed in response to a question with four response options.

Other measures

Demographic characteristics: age, gender, level of education and geographical region of residence. Employment status, planned to be included, was omitted due to a technical error.

Statistical analyses

A detailed statistical analysis plan is available on the Open Science Framework, specified prior to receipt of the data https://osf.io/tjwz8/ *Study 2*. Binary logistic regression was used to assess the impact of test type (immunity/antibody) and result type (passport/certificate/test) on the odds of believing the antibody test result means there is no risk of future infection. An interaction term was included in the model (16). The analysis was repeated adjusting for age (including a quadratic function to model a non-linear relationship), gender, education and region based on prior results showing these are predictors of risk beliefs.

Binary logistic regressions were run (as above) for the secondary outcomes: intention to wash hands less, intention to engage less in social distancing and intention to undergo the test. Unadjusted and adjusted odds ratios and 95% confidence intervals are reported. Logistic regression was run to assess the extent to which intentions to engage in less frequent handwashing or social distancing measures is predicted by perceiving the test result to mean no risk of being infected in the future by coronavirus.

As only a very small proportion of participants gave a 'zero' response on the sliding scale of future risk, we used a linear regression model to examine this outcome, rather than a binary (zero vs. other) logistic regression as pre-specified in the analysis plan.

Procedure

For peer review only - http://bmjopen.bmj.com/site/about/guidelines.xhtml

Data were collected using an online survey platform, Predictiv. Upon entry to the study, participants were informed that they were to be asked some questions about coronavirus and that it would take about five minutes to complete. Participants were then shown one of six brief descriptions of an antibody test for coronavirus (see S1 for full text for each of the six descriptions). They were then asked five questions, assessing the primary and secondary outcomes. Participants' demographic characteristics were accessed from the survey platform.

Results

Sample characteristics

The sample comprised 606 women and 598 men with a median age of 36 years. Around a quarter had some graduate-level education (24.2%) and there was good representation of all UK regions (see Table 1). Distribution of sample characteristics by exposure group is shown in Table 1.

Descriptive statistics

Primary outcome

Responses to the five outcome questions for the whole sample and by experimental group are shown in Table 2. Overall, 14.5% of respondents [95% CI: 12.5 to 16.6] interpreted the test result as meaning they had no risk of future infection. Over half (57.8% [95% CI: 55.0 to 60.1]) correctly interpreted the test result as meaning that their future risk of coronavirus was 'lower'.

Secondary outcomes

Perceived level of future risk (on a scale of 0 to 100%) showed a complex, trimodal distribution. The median was 35% with an interquartile range from 18% to 51%. Only 5% of respondents put their risk at 0%. Overall, 63% put their risk below 50%. 10% put their risk at 50%, which was the modal response. 24% put their risk at greater than 50%, but below 100%. 3% of respondents put their risk at 100%: that is, they said they were certain to contract the virus.

On the behavioural outcomes, 4.9% [95% CI: 3.8 to 6.3] said they would wash their hands less frequently than now if they received a positive result while 19.7% [95% CI: 17.5 to 22.1] said they would be less inclined to avoid physical contact with others outside the home. Intentions to have the test if offered were high, with 56.1%

For peer review only - http://bmjopen.bmj.com/site/about/guidelines.xhtml

[95% CI: 53.2 to 58.9] saying they would definitely, and 29.2% [95% CI: 26.6 to 31.8] saying they would probably have the test if offered today.

For peer review only

For peer review only - http://bmjopen.bmj.com/site/about/guidelines.xhtml

Table 1 – Demographic characteristics of participants by experimental group (n=1204)

Characteristics	All (n=1204)	Immunity Passport (n=187)	Immunity Certificate (n=235)	Immunity Test (n=179)	Antibody Passport (n=219)	Antibody Certificate (n=209)	Antibody Test (n=175)
Gender [n (%)]							
Female	606 (50.3)	86 (46.0)	126 (53.6)	94 (52.5)	112 (51.1)	98 (46.9)	90 (51.4)
Male	598 (49.7)	101 (54.0)	109 (46.4)	85 (47.5)	107 (48.9)	111 (53.1)	85 (48.6)
Age [median (interquartile range)]	36 (32)	35 (34)	36 (34)	36 (31)	38 (32)	34 (27)	36 (31)
Education [n (%)]							
Below degree	888 (73.8)	139 (74.3)	177 (75.3)	126 (70.4)	165 (75.3)	150 (71.8)	131 (74.9)
Degree or above	291 (24.2)	45 (24.1)	52 (22.1)	49 (27.4)	49 (22.4)	55 (26.3)	41 (23.4)
Missing	25 (2.1)	3 (1.6)	6 (2.6)	4 (2.2)	5 (2.3)	4 (1.9)	3 (1.7)
UK region [n (%)]							
England – London	160 (13.3)	24 (12.8)	33 (14.0)	27 (15.1)	25 (11.4)	27 (12.9)	24 (13.7)
England – Midlands	189 (15.7)	38 (20.3)	34 (14.5)	24 (13.4)	30 (13.7)	32 (15.3)	31 (17.7)
England – South & East	373 (31.0)	48 (25.7)	74 (31.5)	51 (28.5)	75 (34.2)	74 (35.4)	51 (29.1)
England – North	308 (25.6)	46 (24.6)	65 (27.7)	50 (27.9)	59 (26.9)	43 (20.6)	45 (25.7)
Scotland/Wales/NI	174 (14.5)	31 (16.6)	29 (12.3)	27 (15.1)	30 (13.7)	33 (15.8)	24 (13.7)

For peer review only

Table 2 – Primary and secondary outcomes by experimental group (n=1204)

	All	Immunity			Antibody		
		Passport	Certificate	Test	Passport	Certificate	Test
	(n=1204)	(n=187)	(n=235)	(n=179)	(n=219)	(n=209)	(n=175)
Perceived meaning of result for future risk [n (%)]							
No risk	174 (14.5)	26 (13.9)	50 (21.3)	39 (21.8)	24 (11.0)	19 (9.1)	16 (9.1)
Lower risk (correct)	697 (57.9)	106 (56.7)	127 (54.0)	92 (51.4)	126 (57.5)	134 (64.1)	112 (64.0)
Average risk	248 (20.6)	40 (21.4)	42 (17.9)	36 (20.1)	54 (24.7)	41 (19.6)	35 (20.0)
Higher risk	85 (7.1)	15 (8.0)	16 (6.8)	12 (6.7)	15 (6.8)	15 (7.2)	12 (6.9)
Perceived absolute risk (0-100) [Mean (SD)]	37.7 (25.2)	40.4 (26.5)	37.0 (25.4)	34.2 (27.1)	39.5 (23.6)	36.9 (25.2)	37.9 (23.1)
Perceived residual risk [n (%)]							
1-100%	1144 (95.0)	179 (95.7)	219 (93.2)	161 (89.9)	214 (97.7)	200 (95.7)	171 (97.7)
0%	60 (5.0)	8 (4.3)	16 (6.8)	18 (10.1)	5 (2.3)	9 (4.3)	4 (2.3)
Intention to wash hands [n (%)]							
Much less than now	13 (1.1)	3 (1.6)	7 (3.0)	0 (0.0)	0 (0.0)	2 (1.0)	1 (0.6)
Less than now	46 (3.8)	6 (3.2)	11 (4.7)	7 (3.9)	8 (3.7)	10 (4.8)	4 (2.3)
Same as now	800 (66.4)	121 (64.7)	159 (67.7)	127 (70.9)	138 (63.0)	143 (68.4)	112 (64.0)
More than now	161 (13.4)	23 (12.3)	26 (11.1)	18 (10.1)	34 (15.5)	29 (13.9)	31 (17.7)
Much more than now	184 (15.3)	34 (18.2)	32 (13.6)	27 (15.1)	39 (17.8)	25 (12.0)	27 (15.4)
Intention to avoid physical contact [n (%)]							
Much less than now	36 (3.0)	2 (1.1)	9 (3.8)	10 (5.6)	4 (1.8)	6 (2.9)	5 (2.9)
Less than now	201 (16.7)	35 (18.7)	48 (20.4)	29 (16.2)	32 (14.6)	33 (15.8)	24 (13.7)
Same as now	642 (53.3)	96 (51.3)	116 (49.4)	96 (53.6)	116 (53.0)	126 (60.3)	92 (52.6)
More than now	178 (14.8)	28 (15.0)	34 (14.5)	21 (11.7)	44 (20.1)	27 (12.9)	24 (13.7)
Much more than now	147 (12.2)	26 (13.9)	28 (11.9)	23 (12.8)	23 (10.5)	17 (8.1)	30 (17.1)
Would you have the test if offered? [n (%)]							
No, definitely not	38 (3.2)	5 (2.7)	14 (6.0)	5 (2.8)	5 (2.3)	4 (1.9)	5 (2.9)
No, probably not	140 (11.6)	24 (12.8)	28 (11.9)	23 (12.8)	23 (10.5)	20 (9.6)	22 (12.6)
Yes, probably	351 (29.2)	54 (28.9)	67 (28.5)	53 (29.6)	62 (28.3)	58 (27.8)	57 (32.6)
Yes, definitely	675 (56.1)	104 (55.6)	126 (53.6)	98 (54.7)	129 (58.9)	127 (60.8)	91 (52.0)

For peer review only

Table 3 – Logistic regression analysis examining impact of Test type and Result type on perception that the test result means 'no risk'

	Proportion answering 'no risk' in each sub-group % (95% CI) (n=1204)	Result means "No Risk" of future infection Odds ratio (95% confidence interval)	
		Mutually adjusted (n=1204)	Adjusted for demographics[1] (n=1179)
Test Type			
Antibody (n=603)	9.8 (7.5-12.4)	Ref	Ref
Immunity (n=601)	19.1 (16.1-22.5)	**2.77 (1.48-5.17)**	**2.91 (1.52-5.55)**
Result Type			
Test (n=354)	15.5 (11.9-19.7)	Ref	Ref
Certificate (n=444)	15.5 (12.3-19.3)	0.99 (0.50-2.00)	0.96 (0.47-1.99)
Passport (n=406)	12.3 (9.3-15.9)	1.22 (0.63-2.38)	1.24 (0.62-2.48)
Test by result interaction			
Certificate by Immunity result		0.98 (0.42-2.27)	1.00 (0.42-2.40)
Passport by Immunity result		0.47 (0.20-1.12)	0.46 (0.19-1.12)

[1]Fully adjusted model includes age (with quadratic term), gender, education level and region (coded as per Table 1)

For peer review only - http://bmjopen.bmj.com/site/about/guidelines.xhtml

Between-group differences in the primary outcome

When Test type (Immunity *vs.* Antibody), Result type (Certificate *vs.* Passport *vs.* Test) and an interaction term were entered into a logistic regression model predicting the belief that the test result meant 'no risk' of future infection (see Table 3), there was a significant effect of Test type, which persisted when we adjusted for demographic factors (age (including a quadratic term), gender, education level and UK region; AOR: 2.91 [95% CI: 1.52 to 5.55]. Those in the 'Immunity' group were more likely to believe the result meant 'no risk' than those in the Antibody group (19.1% [95% CI: 16.1 to 22.5] *vs.* 9.8% [95% CI: 7.5 to 12.4]) [Figure 1]. There was no significant effect of result type and no significant interaction.

Between-group differences in secondary outcomes

We analysed the continuous measures of future perceived risk of infection using a linear model (ANOVA) with two levels for test type, three levels for result type, and an interaction term. Overall, there was no significant effect: $F_{5,1198} = 1.46$, $p = 0.20$, adjusted $R^2 < 1\%$. We repeated the analysis adjusting for demographic factors as covariates. Overall, there was a significant effect: $F_{13,1165} = 1.88$, $p = 0.03$, adjusted $R^2 = 1\%$. This was because of a significant effect of age: as age increased, perceived risk decreased. There remained no significant effect of the experimental variables.

Logistic regression analyses examining the impact of Test type, Result type and their interaction on intentions to wash hands and avoid physical contact less frequently and on willingness to have the test are shown in Supplementary tables 1 and 2. Neither Test type, Result type nor their interaction were significantly associated with these behavioural outcomes.

Association between test result meaning 'no risk' and behavioural intentions

Logistic regression analyses were used to examine belief that the result meant 'no risk' as a predictor of intention to wash hands and avoid physical contact less frequently, given a positive result (see Figure 2 and Supplementary table 3). In analyses adjusting for demographic factors, those who believed there was no residual risk were at increased odds of intending to wash their hands less (AOR: 2.32 [95% CI: 1.25 to 4.28]). The association with intentions to physically distance outside the home was not significant (AOR: 1.37 [95% CI: 0.93 to 2.03]).

For peer review only - http://bmjopen.bmj.com/site/about/guidelines.xhtml

For peer review only

For peer review only - http://bmjopen.bmj.com/site/about/guidelines.xhtml

Discussion

Using the term Immunity – as opposed to Antibody – to describe antibody tests for SARS-CoV-2 doubled the proportion who erroneously perceived they would have no risk of becoming infected with the virus in the future if they were given an antibody-positive test result, from 9.8% for Antibody to 19.1% for Immunity (AOR: 2.91 [95% CI: 1.52 to 5.55]). Using the terms Passport, Certificate or Test to describe the results had no significant effect on risk perception (AOR: 0.96 [95% CI: 0.47-1.99] for Certificate and 1.24 [0.62-2.48] for Passport, compared with Test). The terms used to describe the test and results had no significant direct impact on intentions to engage in the protective behaviours of handwashing or physical distancing. However, across conditions, a greater proportion of those perceiving the result to mean no risk intended to wash their hands less often (10.3% [95% CI: 6.3 to 15.9]) compared with 4.0% who understood there was a residual risk (4.3% [95% CI: 2.8 to 5.3]).

These was no significant association with intended frequency of avoiding physical contact with others outside of the home. Interest in undergoing the test was high – with 85.2% saying they would probably or definitely have it if offered – and was unaffected by the terms used to describe the tests.

This study was designed to test two hypotheses, providing strong support for the first, that describing a test indicating the presence of antibodies using the term Immunity (vs Antibody) increases the likelihood that those with this test result erroneously perceive they have no risk of becoming infected in the future with coronavirus. This likely reflects a certainty about risk of future infection implicit in lay understandings of the term immunity that is not implied by the term antibody (17). Qualitative studies could explore this and other potential mechanisms for the effect observed.

The results of this study did not support the second hypothesis that describing test results as Passports or Certificates increases the likelihood that those with this test result erroneously perceive they have no risk of becoming infected in the future with coronavirus. This does not mean that these terms are unproblematic however, only that they did not influence the specific perceptions that we explored. Qualitative studies are warranted to understand the broader meanings these terms have in the context of testing for antibodies for SARS-CoV-2 and other contexts.

For peer review only - http://bmjopen.bmj.com/site/about/guidelines.xhtml

Responses on the sliding scale of future risk showed a high variability and were largely unexplained by the experimental intervention or other variables measured. This may point to considerable uncertainty in the public as to how to interpret test results. It also likely reflects the well-described tendency of people to use a 50% response to indicate uncertainty rather than a true judgement of probability (18). We also saw that about a quarter of respondents on the first question stated their risk was "average" or "higher". This may point to considerable uncertainty in the public as to how to interpret test results Use of the top end of the scale is hard to interpret but may either reflect a failure to read the information carefully and therefore a misunderstanding of the meaning of the result, or participants' using information beyond the experiment to assess their risk and not adequately considering the hypothetical test result when making their response.

While we found no evidence for a direct effect on protective behaviours of the terms used to describe antibody tests results, there was indirect evidence that perceiving no risk of future infection might reduce frequency of handwashing. This finding is tentative, given it is based on behavioural intentions in response to a hypothetical antibody-positive result. Nonetheless the potential for antibody testing to increase viral transmission must be considered alongside the potential benefits the tests might have in allowing the easing of lockdown restrictions. Clear communication about the ongoing need for handwashing, in particular, will be essential and raising public awareness of the main mechanisms through which SARS-CoV-2 is transmitted – through air and surfaces – might help improve adherence. This, in addition to acknowledgment of the imperfect nature of the tests, will give the public a more accurate representation of the meaning and implications of an antibody test result and a better understanding of how to reduce the risk of transmission. Such communications need to emphasise that transmission can occur through contact regardless of antibody status. Such communications also need to be rigorously evaluated to ensure their effectiveness at communicating these points both to those undergoing antibody tests as well as to general populations that are now having to learn to live with SARS-CoV-2.

Strengths and Limitations

This study provides the first experimental evidence for the potentially adverse impact on risk perceptions and protective behaviours of commonly used terms to describe SARS-CoV-2 antibody tests and their results. As such, it provides timely evidence to inform policy and research to mitigate these effects to realise the potential benefits of such tests.

The study has several limitations. First, participants were responding to a hypothetical test and asked to imagine that they had received a test result that had detected antibodies. Findings from such studies can generalise to clinical settings (19,20) but some caution is warranted.

For peer review only - http://bmjopen.bmj.com/site/about/guidelines.xhtml

Second, the protective behaviours of handwashing and physical distancing were measured using single items assessing behavioural intentions following a hypothetical test result.

Third, the sample size was insufficient to detect effect sizes that could be important at a population level. It is possible, for example, that the use of the terms Certificate or Passport might impact on risk perception, but the current study lacked the power to detect this.

Fourth, while quotas were used to achieve a sample broadly representative of the UK population, research panels are not representative of the general population (21,22). We found no evidence that the impact of the interventions in this study was modified by demographic characteristics of the participants, providing some reassurance about the generalisability of results across age groups, gender, educational level and geographical region of the UK.

Implications for research and policy

The results of this study have several implications for research and policy. The effectiveness of antibody tests for SARS-CoV-2 will depend not only on the extent and duration of any immunity conferred and the performance of a test, but also upon a good understanding of the meaning of tests results among those offered them. First, the use of the term Immunity should be avoided in phrases to describe antibody tests, whether described as Passports, Certificates or Tests. Second, research is needed to evaluate different ways of informing those offered tests and receiving tests results to minimise the proportion erroneously perceiving an antibody-positive test result to mean no risk of becoming infected with the virus. It should also focus on maximising understanding that – regardless of antibody-status – anyone can indirectly transmit the virus by touching a contaminated surface and infecting the next surface they touch. Hand washing or sanitising therefore need to remain frequent. Research is also needed with those undergoing actual tests, powered to detect effects judged meaningful in the context of a population-based testing programme and involving measures of actual behaviour.

Conclusion

Interest in SARS-CoV-2 antibody testing is high – across many countries, employers and populations. While such testing could contribute to wider strategies to ease lock-down restrictions, their use may have an adverse impact on transmission-related behaviour. This appears to vary with the way the tests are described. Using the term Immunity (vs Antibody) to describe antibody tests increases the proportion of people believing that an antibody-positive result means they have no future risk of coronavirus, a perception that may be associated with less frequent handwashing and hence increased risk of transmission.

For peer review only

For peer review only

For peer review only - http://bmjopen.bmj.com/site/about/guidelines.xhtml

Figure 1 **Proportion believing an antibody-positive test result means 'no risk' of future infection** *Error bars show 95% confidence intervals*

See separate file for Figure 1

Figure 2 **Perceived meaning of an antibody-positive test result for future risk and intentions to reduce frequency of hand washing** *Error bars show 95% confidence intervals*

See separate file for Figure 2

For peer review only

For peer review only - http://bmjopen.bmj.com/site/about/guidelines.xhtml

References

1. Hale T, Webster S, Petherick A, Phillips T, Kira B. *Oxford COVID-19 Government Response Tracker*, Blavatnik School of Government [Internet]. 2020. Available from: https://www.bsg.ox.ac.uk/research/research-projects/coronavirus-government-response-tracker

2. World Health Organization. *Critical preparedness, readiness and response actions for COVID-19*. World Health Organization [Internet]. 2020. (Interim Guidance 22 March 2020). Report No.: WHO/2019-nCoV/Community_Actions/2020.3. Available from: https://www.who.int/publications-detail/critical-preparedness-readiness-and-response-actions-for-covid-19

3. Studdert DM, Hall MA. Disease Control, Civil Liberties, and Mass Testing — Calibrating Restrictions during the Covid-19 Pandemic. *N Engl J Med* [Internet]. 2020 Apr 9. Available from: https://doi.org/10.1056/NEJMp2007637

4. Proctor K, Sample I, Oltermann P. 'Immunity passports' could speed up return to work after Covid-19. *The Guardian* [Internet]. 2020 Mar 30; Available from: https://www.theguardian.com/world/2020/mar/30/immunity-passports-could-speed-up-return-to-work-after-covid-19

5. Slotnick D. Delta's CEO said he would support an 'immunity passport' program or other steps to jumpstart travel as the airline reports its first quarterly loss in more than 5 years. *Business Insider* [Internet]. Available from: https://www.businessinsider.com/delta-first-quarter-coronavirus-air-travel-outlook-after-the-pandemic-2020-4

6. Merrick R. People could be given coronavirus 'immunity certificates' to leave lockdown early. *The Independent* [Internet]. 2020 Apr 2. Available from: https://www.independent.co.uk/news/uk/politics/coronavirus-uk-immunity-certificate-boris-johnson-test-germany-a9442211.html

7. Giugliano F. Mass Coronavirus Antibody Tests Have Serious Limits. *Bloomberg.com* [Internet]. 2020 Apr 24. Available from: https://www.bloomberg.com/opinion/articles/2020-04-24/coronavirus-mass-covid-19-antibody-tests-have-serious-limits

8. Forgey Q. Fauci: Coronavirus immunity cards for Americans are 'being discussed'. *POLITICO* [Internet]. Available from: https://www.politico.com/news/2020/04/10/fauci-coronavirus-immunity-cards-for-americans-are-being-discussed-178784

9. Sherwood D. Chile to push ahead with coronavirus 'release certificates' despite WHO warning. *Reuters* [Internet]. 2020 Apr 26. Available from: https://www.reuters.com/article/us-health-coronavirus-chile-idUSKCN2280NW

10. Adams ER, Anand R, Andersson MI, Auckland K, Baillie JK, Barnes E, et al. Evaluation of antibody testing for SARS-Cov-2 using ELISA and lateral flow immunoassays. MedRxiv Prepr. 2020 Jan 1;2020.04.15.20066407.

For peer review only - http://bmjopen.bmj.com/site/about/guidelines.xhtml

11. Altmann DM, Douek DC, Boyton RJ. What policy makers need to know about COVID-19 protective immunity. *The Lancet* [Internet]. 2020 Apr 27. Available from: https://www.thelancet.com/journals/lancet/article/PIIS0140-6736(20)30985-5/abstract

12. Loong T-W. Understanding sensitivity and specificity with the right side of the brain. *BMJ*. 2003;327(7417):716–719.

13. Marteau T, Saidi G, Goodburn S, Lawton J, Michie S, Bobrow M. Numbers or words? A randomized controlled trial of presenting screen negative results to pregnant women. Published in affiliation with the International Society for Prenatal Diagnosis. *Prenat Diagn* 2000209714-718.

14. Marteau TM, Senior V, Sasieni P. Women's understanding of a "normal smear test result": experimental questionnaire based study. *BMJ*. 2001;322(7285):526–528.

15. World Health Organization. *'Immunity passports' in the context of COVID-19* [Internet]. World Health Organization. Report No.: WHO/2019-nCoV/Sci_Brief/Immunity_passport/2020.1. Available from: https://www.who.int/publications-detail/immunity-passports-in-the-context-of-covid-19

16. Jaki T, Vasileiou D. Factorial versus multi-arm multi-stage designs for clinical trials with multiple treatments. *Stat Med*. 2017 Feb 20;36(4):563–80.

17. Lohm D, Davis M, Flowers P, Stephenson N. 'Fuzzy' virus: indeterminate influenza biology, diagnosis and surveillance in the risk ontologies of the general public in time of pandemics. *Health Risk Soc*. 2015 Feb 17;17(2):115–31.

18. de Bruin WB, Fischhoff B, Millstein SG, Halpern-Felsher BL. Verbal and Numerical Expressions of Probability: 'It's a Fifty-Fifty Chance'. *Organ Behav Hum Decis Process*. 2000 Jan;81(1):115–31.

19. Wright AJ, Takeichi C, Whitwell SCL, Hankins M, Marteau TM. The impact of genetic testing for Crohn's disease, risk magnitude and graphical format on motivation to stop smoking: an experimental analogue study. *Clin Genet*. 2008 Apr;73(4):306–14.

20. Hollands GJ, Whitwell SCL, Parker RA, Prescott NJ, Forbes A, Sanderson J, et al. Effect of communicating DNA based risk assessments for Crohn's disease on smoking cessation: randomised controlled trial. BMJ. 2012 Jul 20;345. Available from: https://www.bmj.com/content/345/bmj.e4708

21. Office for National Statistics. *Internet users, UK:2019*. Office for National Statistics; 2019 May. Available from: https://www.ons.gov.uk/businessindustryandtrade/itandinternetindustry/bulletins/internetusers/2019

22. Wright KB. Researching Internet-Based Populations: Advantages and Disadvantages of Online Survey Research, Online Questionnaire Authoring Software Packages, and Web Survey Services. *J Comput-Mediat Commun*. 2005;10(3).

For peer review only - http://bmjopen.bmj.com/site/about/guidelines.xhtml

Funding

Data collection for this study was funded by a block UK government grant to the Behavioural Insights Team. JW is funded by a career development fellowship from Cancer Research UK (ref C7492/A17219). GJR is funded by the National Institute for Health Research Health Protection Research Unit (NIHR HPRU) in Emergency Preparedness and Response at King's College London in partnership with Public Health England (PHE), in collaboration with the University of East Anglia. The views expressed in this paper are those of the authors and not necessarily those of UK government, Cancer Research UK, NIHR or Public Health England.

Competing interests

All authors have completed the Unified Competing Interest form (available on request from the corresponding author) and declare: no support from any organisation for the submitted work; and no financial relationships with any organisations that might have an interest in the submitted work in the previous three years. HWWP declares consultancy fees from Babylon Health; all authors declare no other relationships or activities that could appear to have influenced the submitted work.

Transparency declaration

The authors affirm that the manuscript is an honest, accurate, and transparent account of the study being reported; that no important aspects of the study have been omitted; and that any discrepancies from the study as originally planned have been explained.

Data sharing statement

Anonymised data will be made available upon reasonable request.

Author contribution statement

The study was conceptualised by TMM, JW & GJR. AM completed data collection. JW & HP analysed the data. All authors contributed to, and approved, the final manuscript.

Acknowledgements

We thank Steve Reicher for comments on an earlier draft of the study protocol

For peer review only

Supplementary Materials

S1 Interventions

Intervention A

Immunity Passport

Scientists are developing tests to see who has already had coronavirus.

No test is 100% effective.

This means that those who test 'positive' would have:

- Lower risk of catching coronavirus in the future - and therefore also
- Lower risk of passing it on to others

Those who test 'positive' would get an immunity passport.

They could return to work early.

Intervention B

Immunity Certificate

Scientists are developing tests to see who has already had coronavirus.

No test is 100% effective.

This means that those who test 'positive' would have:

- Lower risk of catching coronavirus in the future - and therefore also
- Lower risk of passing it on to others

Those who test 'positive' would get an immunity certificate.

They could return to work early.

For peer review only - http://bmjopen.bmj.com/site/about/guidelines.xhtml

Intervention C

Immunity Test

Scientists are developing tests to see who has already had coronavirus.

No test is 100% effective.

This means that those who test 'positive' would have:

- Lower risk of catching coronavirus in the future - and therefore also
- Lower risk of passing it on to others

Those who test 'positive' would get a result showing immunity.

They could return to work early.

Intervention D

Antibody Passport

Scientists are developing tests to see who has already had coronavirus.

No test is 100% effective.

This means that those who test 'positive' would have:

- Lower risk of catching coronavirus in the future - and therefore also
- Lower risk of passing it on to others

Those who test 'positive' would get an antibody passport.

They could return to work early.

Intervention E

Antibody Certificate

Scientists are developing tests to see who has already had coronavirus.

No test is 100% effective.

This means that those who test 'positive' would have:

- Lower risk of catching coronavirus in the future - and therefore also
- Lower risk of passing it on to others

Those who test 'positive' would get an antibody certificate.

They could return to work early.

For peer review only - http://bmjopen.bmj.com/site/about/guidelines.xhtml

Intervention F

Antibody Test

Scientists are developing tests to see who has already had coronavirus.

No test is 100% effective.

This means that those who test 'positive' would have:

- Lower risk of catching coronavirus in the future - and therefore also
- Lower risk of passing it on to others

Those who test 'positive' would get a result showing antibodies.

They could return to work early.

For peer review only

For peer review only - http://bmjopen.bmj.com/site/about/guidelines.xhtml

S2. Measures

Primary outcome

I Imagine you were given *[an immunity passport/an immunity certificate/a result showing immunity/an antibody passport/an antibody certificate/a result showing antibodies]*

which one of the following statements is true*: [order of response options to be randomised]*

1. I have no risk of catching coronavirus in the future
2. I have a lower risk of catching coronavirus in the future
3. I have an average risk of catching coronavirus in the future
4. I have a higher risk of catching coronavirus in the future

Secondary outcomes

II Imagine you were given *[an immunity passport/an immunity certificate/a result showing immunity/an antibody passport/an antibody certificate/a result showing antibodies]*

how likely is it that you will get coronavirus at some point in the future?

Please answer on a scale from 0 % to 100% where 0% means no chance and 100% means certain.

0% --100%

III Imagine that you were given *[an immunity passport/an immunity certificate/a result showing immunity/an antibody passport/an antibody certificate/a result showing antibodies]*

would you wash your hands with soap and water or sanitiser:

1. Much more than now
2. More than now
3. Same as now
4. Less than now
5. Much less than now

IV Imagine you were given *[an immunity passport/an immunity certificate/a result showing immunity/an antibody passport/an antibody certificate/a result showing antibodies]*

would you avoid physical contact with others outside of your home:

1. Much more than now
2. More than now
3. Same as now
4. Less than now
5. Much less than now

V If you were offered a test today by the NHS to check whether you have ever had coronavirus, would you have it?

1. Yes, definitely
2. Yes, probably
3. No, probably not
4. No, definitely not

For peer review only - http://bmjopen.bmj.com/site/about/guidelines.xhtml

For peer review only

Supplementary Table 1 – Logistic regression analysis examining impact of Test type and Result type on protective behaviours

	Intend to wash hands less			Intend to avoid contact less		
	% responding less/much less in each sub-group % (95% CI)	Mutually adjusted (n=1204)	Adjusted for demographics[1] (n=1179)	% responding less/much less in each sub-group % (95% CI)	Mutually adjusted (n=1204)	Adjusted for demographics[1] (n=1179)
Test Type						
Antibody (n=603)	4.1 (2.7-6.1)	Ref	Ref	17.2 (104)	Ref	Ref
Immunity (n=601)	5.7 (4.0-7.8)	1.38 (0.43-4.45)	1.62 (0.46-5.73)	22.1 (133)	1.40 (0.82-2.39)	1.46 (0.85-2.50)
Result Type						
Test (n=354)	3.4 (1.8-5.9)	Ref	Ref	19.2 (68)	Ref	Ref
Certificate (n=444)	6.8 (4.6-9.5)	2.07 (0.72-6.00)	2.47 (0.77-7.96)	21.6 (96)	1.16 (0.68-1.96)	1.14 (0.67-1.96)
Passport (n=406)	4.2 (2.5-6.6)	1.29 (0.41-4.01)	1.79 (0.52-6.16)	18.0 (73)	0.99 (0.58-1.69)	1.02 (0.59-1.76)
Test by Result interaction						
Certificate by Immunity result		0.98 (0.25-3.95)	0.80 (0.18-3.56)		1.00 (0.49-2.01)	0.94 (0.46-1.93)
Passport by Immunity result		0.96 (0.21-4.40)	0.58 (0.11-2.98)		0.89 (0.43-1.87)	0.84 (0.40-1.78)

[1]Fully adjusted model includes age (with quadratic term), gender, education level and region (coded as per Table 1)

For peer review only

Supplementary Table 2 – Logistic regression examining impact of Test and Result type on intention to have the test

		Do not intend to have the test	
	Proportion who would not have test in each sub-group % (95% CI)	Mutually adjusted (n=1204)	Adjusted for demographics[1] (n=1179)
Test Type			
Antibody (n=603)	13.1 (10.5-16.1)	Ref	Ref
Immunity (n=601)	16.5 (13.6-19.7)	1.02 (0.57-1.81)	0.97 (0.54-1.74)
Result Type			
Test (n=354)	15.5 (11.9-19.7)	Ref	Ref
Certificate (n=444)	14.9 (11.7-18.5)	0.71 (0.39-1.28)	0.63 (0.34-1.15)
Passport (n=406)	14.0 (10.8-17.8)	0.80 (0.54-1.42)	0.82 (0.46-1.47)
Test by Result interaction			
Certificate by Immunity result		1.65 (0.75-3.63)	1.91 (0.84-4.31)
Passport by Immunity result		1.23 (0.55-2.75)	1.22 (0.54-2.78)

[1]Fully adjusted model includes age (with quadratic term), gender, education level and region (coded as per Table 1)

For peer review only

Supplementary Table 3 – Logistic regression analysis examining impact of perceived risk on anticipated behaviour

	Intend to wash hands less			Intend to avoid contact less		
	% responding less/much less in each sub-group % (95% CI)	Unadjusted (n=1204)	Adjusted for demographics[1] (n=1179)	% responding less/much less in each sub-group % (95% CI)	Unadjusted (n=1204)	Adjusted for demographics[1] (n=1179)
Perceived meaning of result						
Some residual risk (n=1030)	4.0 (2.8-5.3)	Ref	Ref	18.8 (16.5-21.4)	Ref	Ref
No residual risk of future infection (n=175)	10.3 (6.3-15.9)	**2.78 (1.56-4.97)**	**2.32 (1.25-4.28)**	24.7 (18.5-31.8)	1.41 (0.97-2.06)	1.37 (0.93-2.03)

[1]Fully adjusted model includes age (with quadratic term), gender, education level and region (coded as per Table 1)

For peer review only

For peer review only - http://bmjopen.bmj.com/site/about/guidelines.xhtml

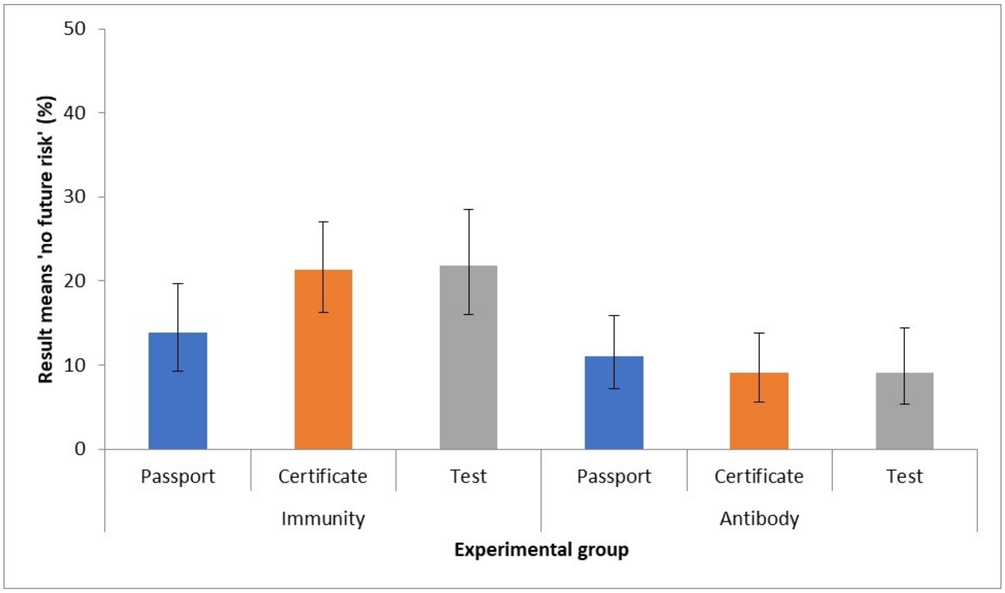

164x96mm (150 x 150 DPI)

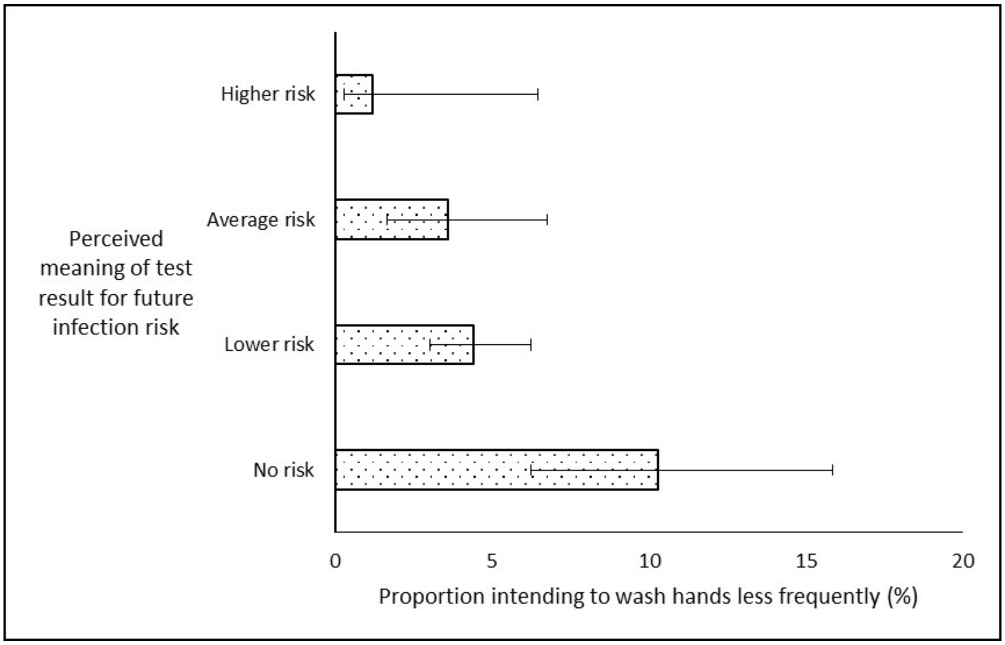

145x92mm (150 x 150 DPI)

“Immunity Passports” for SARS-CoV-2: an online experimental study of the impact of antibody test terminology on perceived risk and behaviour

Journal:	*BMJ Open*
Manuscript ID	bmjopen-2020-040448.R1
Article Type:	Original research
Date Submitted by the Author:	17-Jun-2020
Complete List of Authors:	Waller, Jo; UCL, Epidemiology and Public Health<br>Rubin, GJ; King\'s College London School of Medical Education<br>Potts, Henry; UCL, Centre for Health Informatics and Multiprofessional Education<br>Mottershaw, Abigail; Behavioural Insights Team<br>Marteau, Theresa; University of Cambridge, Behaviour and Health Research Unit
**Primary Subject Heading**:	Public health
Secondary Subject Heading:	Infectious diseases
Keywords:	Public health < INFECTIOUS DISEASES, PUBLIC HEALTH, IMMUNOLOGY

SCHOLARONE™
Manuscripts

I, the Submitting Author has the right to grant and does grant on behalf of all authors of the Work (as defined in the below author licence), an exclusive licence and/or a non-exclusive licence for contributions from authors who are: i) UK Crown employees; ii) where BMJ has agreed a CC-BY licence shall apply, and/or iii) in accordance with the terms applicable for US Federal Government officers or employees acting as part of their official duties; on a worldwide, perpetual, irrevocable, royalty-free basis to BMJ Publishing Group Ltd ("BMJ") its licensees and where the relevant Journal is co-owned by BMJ to the co-owners of the Journal, to publish the Work in this journal and any other BMJ products and to exploit all rights, as set out in our licence.

The Submitting Author accepts and understands that any supply made under these terms is made by BMJ to the Submitting Author unless you are acting as an employee on behalf of your employer or a postgraduate student of an affiliated institution which is paying any applicable article publishing charge ("APC") for Open Access articles. Where the Submitting Author wishes to make the Work available on an Open Access basis (and intends to pay the relevant APC), the terms of reuse of such Open Access shall be governed by a Creative Commons licence – details of these licences and which Creative Commons licence will apply to this Work are set out in our licence referred to above.

Other than as permitted in any relevant BMJ Author's Self Archiving Policies, I confirm this Work has not been accepted for publication elsewhere, is not being considered for publication elsewhere and does not duplicate material already published. I confirm all authors consent to publication of this Work and authorise the granting of this licence.

For peer review only

DRAFT Revised MS BMJ Open 15 June 2020

"Immunity Passports" for SARS-CoV-2: an online experimental study of the impact of antibody test terminology on perceived risk and behaviour

Jo Waller PhD[1], G. James Rubin PhD[2], Henry W. W. Potts PhD[3], Abi Mottershaw[4], Theresa M Marteau PhD[5]*

[1] Jo Waller, Reader in Cancer Behavioural Science, School of Cancer and Pharmaceutical Sciences, King's College London, Guy's Hospital, Great Maze Pond, London, SE1 9RT.

[2] G. James Rubin, Reader in the Psychology of Emerging Health Risks. Department of Psychological Medicine, King's College London, Weston Education Centre, Cutcombe Road, London, SE5 9RJ.

[3] Henry W. W. Potts, Associate Professor, Institute of Health Informatics, University College London, 222 Euston Road London, NW1 2DA.

[4]Abigail L. Mottershaw, Online Experiments Lead. Behavioural Insights Team, 4 Matthew Parker Street, London, SW1H 9NP.

*[5] Theresa M Marteau, Director of Research, Behaviour and Health Research Unit, University of Cambridge, Institute of Public Health, Forvie Site, Robinson Way, Cambridge, CB2 0SR.

* corresponding author: tm388@cam.ac.uk

For peer review only

DRAFT Revised MS BMJ Open 15 June 2020

Objective: To assess the impact of describing an antibody-positive test result using the terms Immunity and Passport or Certificate, alone or in combination, on perceived risk of becoming infected with SARS-CoV-2 and protective behaviours.

Design: 2 × 3 experimental design.

Setting: Online

Participants: 1,204 adults from a UK research panel.

Intervention: Participants were randomised to receive one of six descriptions of an antibody test and results showing SARS-CoV-2 antibodies, differing in the terms describing the type of test (Immunity *vs* Antibody) and the test result (Passport *vs* Certificate *vs* Test).

Main outcome measures: Primary outcome: proportion of participants perceiving no risk of infection with SARS-CoV-2 given an antibody positive test result. Other outcomes include: intended changes to frequency of handwashing and physical distancing.

Results: When using the term Immunity (vs Antibody), 19.1% of participants [95% CI: 16.1 to 22.5] (vs 9.8% [95% CI: 7.5 to 12.4]) perceived no risk of catching coronavirus given an antibody-positive test result (AOR: 2.91 [95% CI: 1.52 to 5.55]). Using the terms Passport or Certificate – as opposed to Test – had no significant effect (AOR: 1.24 [95% CI: 0.62 to 2.48] and AOR: 0.96 [95% CI: 0.47 to 1.99] respectively). There was no significant interaction between the effects of the test and result terminology. Across groups, perceiving no risk of infection was associated with an intention to wash hands less frequently (AOR: 2.32 [95% CI: 1.25 to 4.28]); there

For peer review only - http://bmjopen.bmj.com/site/about/guidelines.xhtml

was no significant association with intended avoidance of physical distancing (AOR: 1.37 [95% CI: 0.93 to 2.03]).

Conclusions: Using the term Immunity (vs Antibody) to describe antibody tests for SARS-CoV-2 increases the proportion of people believing that an antibody-positive result means they have no risk of catching coronavirus in the future, a perception that may be associated with less frequent handwashing.

Key words: SARS-CoV-2; COVID-19; antibody testing; immunity; passport; certificate; risk perception; protective behaviours

Study registration: Open Science Framework: https://osf.io/tjwz8/files/

For peer review only

DRAFT Revised MS BMJ Open 15 June 2020

Strengths and limitations of this study

- This study provides the first experimental evidence for the potentially adverse impact on risk perceptions and protective behaviours of using the term Immunity – as opposed to Antibody - to describe SARS-CoV-2 antibody tests and their results.
- It provides timely evidence to inform policy and research to mitigate these effects to realise the potential benefits of such tests.
- Participants were responding to a hypothetical test and asked to imagine that they had received a test result that had detected antibodies.
- Protective behaviours of handwashing and physical distancing were measured using single items assessing behavioural intentions.

For peer review only

For peer review only - http://bmjopen.bmj.com/site/about/guidelines.xhtml

Introduction

At the height of the first wave of the COVID-19 pandemic, about a third of the world's population is estimated to have been in lockdown, with all but essential workers largely confined to home (1).

Without an effective treatment or vaccine, testing for infection combined with contact tracing and isolation will be central to effective strategies to ease populations out of lockdown while keeping the basic reproduction number (R_0) below one (2).

Testing for antibodies to SARS-CoV-2 is a possible complement to testing for active infection to identify those who have developed antibodies to the virus and so may be able to return to work and other activities without significantly increasing transmission rates (3). These tests have been variously described in the media as Immunity Passports (4,5), Immunity Certificates (6,7) Immunity Cards (8) and Release Certificates (9). Unfortunately, the use of these terms implies a certainty unmatched by current evidence about antibody tests (10).

Uncertainties inherent in tests for antibodies to SARS-CoV-2 include the extent and duration of immunity conferred (11). They also include the uncertainties inherent in any test regarding the proportion of those who would be correctly identified. This depends upon the test performance – its sensitivity and specificity – as well as the population prevalence of the tested condition (12). Given these uncertainties, those who receive a test result indicating the presence of antibodies will have a residual risk of becoming infected by SARS-CoV-2 in the future.

Understanding that there is this residual risk – albeit one that is difficult to quantify at present – will be important to minimise transmission that could arise from those receiving "antibody positive" test results. If people testing positive perceive that they

For peer review only - http://bmjopen.bmj.com/site/about/guidelines.xhtml

have no risk of becoming infected by the virus, they may ignore any future symptoms of infection and facilitate transmission if they fail to self-isolate appropriately. Such a perception may also over-generalise to a belief that they are unable to transmit infection through contact with contaminated surfaces. Regardless of antibody status, all individuals can indirectly transmit the virus between surfaces by touch. Hand washing or sanitising therefore need to remain frequent.

Evidence from other testing programmes suggests that interpreting a low risk result to mean no risk can be reduced by verbal and numerical expressions of residual risk when presenting test results (13,14). But even before testing programmes are in place, the terms commonly used to describe these tests – Immunity Passport or Certificates – may inadvertently be fuelling a misplaced sense of certainty about their results. It is unknown whether describing these tests as being for immunity – as opposed to antibodies – or their results as passports or certificates increases misunderstanding of the residual risk inherent in an antibody-positive test result and thereby reducing adherence to protective behaviours and increasing risk of transmission (15).

This study was designed to test two hypotheses: describing a test indicating the presence of antibodies using the term Immunity (vs Antibody), and describing test results as Passports or Certificates (vs Test), increases the likelihood that those with this test result erroneously perceive they have no risk of becoming infected in the future with coronavirus.

Methods

Ethical approval for this study was granted by the King's College London Research Ethics Committee (reference: MRA-19/20-18685).

For peer review only - http://bmjopen.bmj.com/site/about/guidelines.xhtml

The protocol was preregistered on the Open Science Framework https://osf.io/tjwz8/ *Study 2*

The statistical analysis plan was pre-specified and uploaded to the Open Science Framework prior to receipt of the data https://osf.io/tjwz8/ *Study 2*

An initial study with similar methods was conducted https://osf.io/tjwz8/ *Study 1* but, due to an error, the intervention was not correctly programmed. This study is therefore not reported.

Design

The study was an online experiment using a 2 × 3 factorial design, with participants randomised, with an equal allocation ratio, to one of six groups varying in the description of an antibody test and a result showing the presence of antibodies. These descriptions differed only in the term used for what was being tested (Immunity *vs* Antibody) and the term used for the test result (Passport *vs* Certificate *vs* Test).

Participants

A quota sample of 1,204 adults was recruited via Predictiv, the Behavioural Insights Team's online experimentation platform (https://www.bi.team/bi-ventures/predictiv/) comprising 500,000 adults in the UK. Quotas were based on age, gender and UK region to achieve a sample broadly representative of the UK population. 1373 clicked on the link to enter the study of whom 1214 subsequently completed the study. Ten were excluded for failing to meet quality checks (e.g. the unique participant identifier was missing, or the participant had entered the survey more than once). Participants were reimbursed in points (equivalent to £1) which could be redeemed in cash, gift vouchers or charitable donations. Participants did not know the topic of the study

For peer review only - http://bmjopen.bmj.com/site/about/guidelines.xhtml

prior to participation. All participants had provided opt-in consent to take part in surveys when they signed up to the online panel. No identifiable data were collected as part of the survey. All data collected by Predictiv are processed in line with their published privacy policy (http://www.predictiv.co.uk/privacy-policy.html).

Patient and public involvement

Due to the rapid nature of this research, the public was not involved in the development of the study.

Power

The sample size was chosen pragmatically without reference to a specific power calculation. We fitted a full linear model with two levels for test type (immunity/antibody), three levels for result type (passport/certificate/test) and an interaction term. Conservatively, we then had an 80% chance of detecting, at a 5% significance level, an increase in the primary outcome measure from 50% in a baseline group to 64% in another group. The power calculation was done using http://powerandsamplesize.com/, taking 50% as the baseline to provide a conservative estimate of power.

Randomisation

Participants were randomised to groups by random number generation. A random number between 1 and 6 was generated for every participant upon entry to the study to determine which description they saw, with each of the six numbers corresponding to one of the six description. As this is based on true randomness, the number of participants within each group can vary due to chance (see Table 1 for numbers in each group).

Intervention

For peer review only - http://bmjopen.bmj.com/site/about/guidelines.xhtml

The intervention comprised a description of antibody testing and test results indicating the presence of antibodies [see Box 1 for one example and S1 for wording of all six descriptions]. These differed across six groups in test name of results indicating the presence of antibodies. All descriptions included the information that the result would mean a lower risk of future infection and transmission, and that people with this result could return to work earlier.

Box Immunity Passport: one of the six descriptions of an antibody-positive test result

Immunity Passport

Scientists are developing tests to see who has already had coronavirus.

No test is 100% effective.

This means that those who test 'positive' would have:

- Lower risk of catching coronavirus in the future, and therefore also
- Lower risk of passing it on to others

Those who test 'positive' would get an immunity passport.

They could return to work early.

Outcomes measures

Wording of the items used for each measure is shown in Supplementary Materials (S2). Response options to all outcome measures are shown in Table 2.

Primary outcome

Proportion of participants perceiving an antibody-positive test result to mean no risk of catching coronavirus in the future, assessed in response to a question with four response options.

Secondary outcomes

For peer review only - http://bmjopen.bmj.com/site/about/guidelines.xhtml

Perceived likelihood of catching coronavirus in the future, assessed on a visual analogue scale from 0% to 100%.

Intention to engage in handwashing less or more frequently than now, given an antibody-positive test result: assessed in response to a question with five response options.

Intention to avoid physical contact with others outside the home more or less frequently than now, given an antibody-positive test result: assessed in response to a question with five response options.

Interest in undergoing the test if offered today: assessed in response to a question with four response options.

Other measures

Demographic characteristics: age, gender, level of education and geographical region of residence. Employment status, planned to be included, was omitted due to a technical error.

Statistical analyses

A detailed statistical analysis plan is available on the Open Science Framework, specified prior to receipt of the data https://osf.io/tjwz8/ *Study 2*. Binary logistic regression was used to assess the impact of test type (immunity/antibody) and result type (passport/certificate/test) on the odds of believing the antibody test result means there is no risk of future infection. An interaction term was included in the model (16). The analysis was repeated adjusting for age (including a quadratic function to model a non-linear relationship), gender, education and region based on prior results showing these are predictors of risk beliefs.

For peer review only

Binary logistic regressions were run (as above) for the secondary outcomes: intention to wash hands less, intention to engage less in social distancing and intention to undergo the test. Unadjusted and adjusted odds ratios and 95% confidence intervals are reported. Logistic regression was run to assess the extent to which intentions to engage in less frequent handwashing or social distancing measures is predicted by perceiving the test result to mean no risk of being infected in the future by coronavirus.

As only a very small proportion of participants gave a 'zero' response on the sliding scale of future risk, we used a linear regression model to examine this outcome, rather than a binary (zero vs. other) logistic regression as pre-specified in the analysis plan.

Procedure

Data were collected using an online survey platform, Predictiv. Upon entry to the study, participants were informed that they were to be asked some questions about coronavirus and that it would take about five minutes to complete. Participants were then shown one of six brief descriptions of an antibody test for coronavirus (see S1 for full text for each of the six descriptions). They were then asked five questions, assessing the primary and secondary outcomes. Participants' demographic characteristics were accessed from the survey platform.

Results

Sample characteristics

The sample comprised 606 women and 598 men with a median age of 36 years. Around a quarter had some graduate-level education (24.2%) and there was good

For peer review only

representation of all UK regions (see Table 1). Distribution of sample characteristics by exposure group is shown in Table 1.

Descriptive statistics

Primary outcome

Responses to the five outcome questions for the whole sample and by experimental group are shown in Table 2. Overall, 14.5% of respondents [95% CI: 12.5 to 16.6] interpreted the test result as meaning they had no risk of future infection. Over half (57.8% [95% CI: 55.0 to 60.1]) correctly interpreted the test result as meaning that their future risk of coronavirus was 'lower'.

Secondary outcomes

Perceived level of future risk (on a scale of 0 to 100%) showed a complex, trimodal distribution. The median was 35% with an interquartile range from 18% to 51%. Only 5% of respondents put their risk at 0%. Overall, 63% put their risk below 50%. 10% put their risk at 50%, which was the modal response. 24% put their risk at greater than 50%, but below 100%. 3% of respondents put their risk at 100%: that is, they said they were certain to contract the virus. There was a lack of consistency between responses to the categorical and the continuous measures of risk. Median responses on the sliding scale were 12.5%, 28.0%, 50.0% and 65.0% in the 'no risk', 'lower risk', 'average risk' and 'higher risk' categories respectively, with responses ranging from 0-100 for all four of these groups. Although this suggests that a proportion of people were responding very differently to the two questions (e.g. 'no risk' on the first outcome and 100% risk on the sliding scale), the increasing median responses with increasing risk category provides some reassurance that most people's responses were broadly consistent across the two measures.

On the behavioural outcomes, 4.9% [95% CI: 3.8 to 6.3] said they would wash their hands less frequently than now if they received a positive result while 19.7% [95%

For peer review only - http://bmjopen.bmj.com/site/about/guidelines.xhtml

CI: 17.5 to 22.1] said they would be less inclined to avoid physical contact with others outside the home. Intentions to have the test if offered were high, with 56.1% [95% CI: 53.2 to 58.9] saying they would definitely, and 29.2% [95% CI: 26.6 to 31.8] saying they would probably have the test if offered today.

Between-group differences in the primary outcome

When Test type (Immunity *vs.* Antibody), Result type (Certificate *vs.* Passport *vs.* Test) and an interaction term were entered into a logistic regression model predicting the belief that the test result meant 'no risk' of future infection (see Table 3), there was a significant effect of Test type, which persisted when we adjusted for demographic factors (age (including a quadratic term), gender, education level and UK region; AOR: 2.91 [95% CI: 1.52 to 5.55]. Those in the 'Immunity' group were more likely to believe the result meant 'no risk' than those in the Antibody group (19.1% [95% CI: 16.1 to 22.5] *vs.* 9.8% [95% CI: 7.5 to 12.4]) [Figure 1]. There was no significant effect of result type and no significant interaction.

Between-group differences in secondary outcomes

We analysed the continuous measures of future perceived risk of infection using a linear model (ANOVA) with two levels for test type, three levels for result type, and an interaction term. Overall, there was no significant effect: $F_{5,1198} = 1.46$, $p = 0.20$, adjusted $R^2 < 1\%$. We repeated the analysis adjusting for demographic factors as covariates. Overall, there was a significant effect: $F_{13,1165} = 1.88$, $p = 0.03$, adjusted $R^2 = 1\%$. This was because of a significant effect of age: as age increased, perceived risk decreased. There remained no significant effect of the experimental variables.

Logistic regression analyses examining the impact of Test type, Result type and their interaction on intentions to wash hands and avoid physical contact less frequently and on willingness to have the test are shown in Supplementary tables 1 and 2. Neither Test type, Result type nor their interaction were significantly associated with these behavioural outcomes.

For peer review only - http://bmjopen.bmj.com/site/about/guidelines.xhtml

DRAFT Revised MS BMJ Open 15 June 2020

For peer review only - http://bmjopen.bmj.com/site/about/guidelines.xhtml

DRAFT Revised MS BMJ Open 15 June 2020

Table 1 – Demographic characteristics of participants by experimental group (n=1204)

Characteristics	All (n=1204)	Immunity Passport (n=187)	Immunity Certificate (n=235)	Immunity Test (n=179)	Antibody Passport (n=219)	Antibody Certificate (n=209)	Antibody Test (n=175)
Gender [n (%)]							
Female	606 (50.3)	86 (46.0)	126 (53.6)	94 (52.5)	112 (51.1)	98 (46.9)	90 (51.4)
Male	598 (49.7)	101 (54.0)	109 (46.4)	85 (47.5)	107 (48.9)	111 (53.1)	85 (48.6)
Age [median (interquartile range)]	36 (32)	35 (34)	36 (34)	36 (31)	38 (32)	34 (27)	36 (31)
Education [n (%)]							
Below degree	888 (73.8)	139 (74.3)	177 (75.3)	126 (70.4)	165 (75.3)	150 (71.8)	131 (74.9)
Degree or above	291 (24.2)	45 (24.1)	52 (22.1)	49 (27.4)	49 (22.4)	55 (26.3)	41 (23.4)
Missing	25 (2.1)	3 (1.6)	6 (2.6)	4 (2.2)	5 (2.3)	4 (1.9)	3 (1.7)
UK region [n (%)]							
England – London	160 (13.3)	24 (12.8)	33 (14.0)	27 (15.1)	25 (11.4)	27 (12.9)	24 (13.7)
England – Midlands	189 (15.7)	38 (20.3)	34 (14.5)	24 (13.4)	30 (13.7)	32 (15.3)	31 (17.7)
England – South & East	373 (31.0)	48 (25.7)	74 (31.5)	51 (28.5)	75 (34.2)	74 (35.4)	51 (29.1)
England – North	308 (25.6)	46 (24.6)	65 (27.7)	50 (27.9)	59 (26.9)	43 (20.6)	45 (25.7)
Scotland/Wales/NI	174 (14.5)	31 (16.6)	29 (12.3)	27 (15.1)	30 (13.7)	33 (15.8)	24 (13.7)

For peer review only - http://bmjopen.bmj.com/site/about/guidelines.xhtml

DRAFT Revised MS BMJ Open 15 June 2020

Table 2 – Primary and secondary outcomes by experimental group (n=1204)

	All (n=1204)	Immunity Passport (n=187)	Immunity Certificate (n=235)	Immunity Test (n=179)	Antibody Passport (n=219)	Antibody Certificate (n=209)	Antibody Test (n=175)
Perceived meaning of result for future risk [n (%)]							
No risk	174 (14.5)	26 (13.9)	50 (21.3)	39 (21.8)	24 (11.0)	19 (9.1)	16 (9.1)
Lower risk (correct)	697 (57.9)	106 (56.7)	127 (54.0)	92 (51.4)	126 (57.5)	134 (64.1)	112 (64.0)
Average risk	248 (20.6)	40 (21.4)	42 (17.9)	36 (20.1)	54 (24.7)	41 (19.6)	35 (20.0)
Higher risk	85 (7.1)	15 (8.0)	16 (6.8)	12 (6.7)	15 (6.8)	15 (7.2)	12 (6.9)
Perceived absolute risk (0-100) [Mean (SD)]	37.7 (25.2)	40.4 (26.5)	37.0 (25.4)	34.2 (27.1)	39.5 (23.6)	36.9 (25.2)	37.9 (23.1)
Perceived residual risk [n (%)]							
1-100%	1144 (95.0)	179 (95.7)	219 (93.2)	161 (89.9)	214 (97.7)	200 (95.7)	171 (97.7)
0%	60 (5.0)	8 (4.3)	16 (6.8)	18 (10.1)	5 (2.3)	9 (4.3)	4 (2.3)
Intention to wash hands [n (%)]							
Much less than now	13 (1.1)	3 (1.6)	7 (3.0)	0 (0.0)	0 (0.0)	2 (1.0)	1 (0.6)
Less than now	46 (3.8)	6 (3.2)	11 (4.7)	7 (3.9)	8 (3.7)	10 (4.8)	4 (2.3)
Same as now	800 (66.4)	121 (64.7)	159 (67.7)	127 (70.9)	138 (63.0)	143 (68.4)	112 (64.0)
More than now	161 (13.4)	23 (12.3)	26 (11.1)	18 (10.1)	34 (15.5)	29 (13.9)	31 (17.7)
Much more than now	184 (15.3)	34 (18.2)	32 (13.6)	27 (15.1)	39 (17.8)	25 (12.0)	27 (15.4)
Intention to avoid physical contact [n (%)]							
Much less than now	36 (3.0)	2 (1.1)	9 (3.8)	10 (5.6)	4 (1.8)	6 (2.9)	5 (2.9)
Less than now	201 (16.7)	35 (18.7)	48 (20.4)	29 (16.2)	32 (14.6)	33 (15.8)	24 (13.7)
Same as now	642 (53.3)	96 (51.3)	116 (49.4)	96 (53.6)	116 (53.0)	126 (60.3)	92 (52.6)
More than now	178 (14.8)	28 (15.0)	34 (14.5)	21 (11.7)	44 (20.1)	27 (12.9)	24 (13.7)
Much more than now	147 (12.2)	26 (13.9)	28 (11.9)	23 (12.8)	23 (10.5)	17 (8.1)	30 (17.1)
Would you have the test if offered? [n (%)]							
No, definitely not	38 (3.2)	5 (2.7)	14 (6.0)	5 (2.8)	5 (2.3)	4 (1.9)	5 (2.9)
No, probably not	140 (11.6)	24 (12.8)	28 (11.9)	23 (12.8)	23 (10.5)	20 (9.6)	22 (12.6)
Yes, probably	351 (29.2)	54 (28.9)	67 (28.5)	53 (29.6)	62 (28.3)	58 (27.8)	57 (32.6)
Yes, definitely	675 (56.1)	104 (55.6)	126 (53.6)	98 (54.7)	129 (58.9)	127 (60.8)	91 (52.0)

For peer review only

Table 3 – Logistic regression analysis examining impact of Test type and Result type on perception that the test result means 'no risk'

	Proportion answering 'no risk' in each sub-group % (95% CI) (n=1204)	Result means "No Risk" of future infection Odds ratio (95% confidence interval)	
		Mutually adjusted (n=1204)	Adjusted for demographics[1] (n=1179)
Test Type			
Antibody (n=603)	9.8 (7.5-12.4)	Ref	Ref
Immunity (n=601)	19.1 (16.1-22.5)	**2.77 (1.48-5.17)**	**2.91 (1.52-5.55)**
Result Type			
Test (n=354)	15.5 (11.9-19.7)	Ref	Ref
Certificate (n=444)	15.5 (12.3-19.3)	0.99 (0.50-2.00)	0.96 (0.47-1.99)
Passport (n=406)	12.3 (9.3-15.9)	1.22 (0.63-2.38)	1.24 (0.62-2.48)
Test by result interaction			
Certificate by Immunity result		0.98 (0.42-2.27)	1.00 (0.42-2.40)
Passport by Immunity result		0.47 (0.20-1.12)	0.46 (0.19-1.12)

[1]Fully adjusted model includes age (with quadratic term), gender, education level and region (coded as per Table 1)

For peer review only - http://bmjopen.bmj.com/site/about/guidelines.xhtml

Association between test result meaning 'no risk' and behavioural intentions

Logistic regression analyses were used to examine belief that the result meant 'no risk' as a predictor of intention to wash hands and avoid physical contact less frequently, given a positive result (see Figure 2 and Supplementary table 3). In analyses adjusting for demographic factors, those who believed there was no residual risk were at increased odds of intending to wash their hands less (AOR: 2.32 [95% CI: 1.25 to 4.28]). The association with intentions to physically distance outside the home was not significant (AOR: 1.37 [95% CI: 0.93 to 2.03]).

Discussion

Using the term Immunity – as opposed to Antibody – to describe antibody tests for SARS-CoV-2 doubled the proportion who erroneously perceived they would have no risk of becoming infected with the virus in the future if they were given an antibody-positive test result, from 9.8% for Antibody to 19.1% for Immunity (AOR: 2.91 [95% CI: 1.52 to 5.55]). Using the terms Passport, Certificate or Test to describe the results had no significant effect on risk perception (AOR: 0.96 [95% CI: 0.47-1.99] for Certificate and 1.24 [0.62-2.48] for Passport, compared with Test). The terms used to describe the test and results had no significant direct impact on intentions to engage in the protective behaviours of handwashing or physical distancing. However, across conditions, a greater proportion of those perceiving the result to mean no risk intended to wash their hands less often (10.3% [95% CI: 6.3 to 15.9]) compared with 4.0% who understood there was a residual risk (4.3% [95% CI: 2.8 to 5.3]).

These was no significant association with intended frequency of avoiding physical contact with others outside of the home. Interest in undergoing the test was high – with 85.2% saying they would probably or definitely have it if offered – and was unaffected by the terms used to describe the tests.

For peer review only - http://bmjopen.bmj.com/site/about/guidelines.xhtml

This study was designed to test two hypotheses, providing strong support for the first, that describing a test indicating the presence of antibodies using the term Immunity (vs Antibody) increases the likelihood that those with this test result erroneously perceive they have no risk of becoming infected in the future with coronavirus. This likely reflects a certainty about risk of future infection implicit in lay understandings of the term immunity that is not implied by the term antibody (17). Qualitative studies could explore this and other potential mechanisms for the effect observed.

The results of this study did not support the second hypothesis that describing test results as Passports or Certificates increases the likelihood that those with this test result erroneously perceive they have no risk of becoming infected in the future with coronavirus. This does not mean that these terms are unproblematic however, only that they did not influence the specific perceptions that we explored. Qualitative studies are warranted to understand the broader meanings these terms have in the context of testing for antibodies for SARS-CoV-2 and other contexts.

Responses on the sliding scale of future risk showed a high variability and were largely unexplained by the experimental intervention or other variables measured. They also showed only broad consistency with responses to the main outcome measure; some participants who believed the result meant 'no risk' then rated their future risk as 100%, and vice versa. This may point to considerable uncertainty in the public as to how to interpret test results. Some participants may have been responding solely on the information provided; others may have been using other information about coronavirus and immunity to make judgements about their own risk. It also likely reflects the well-described tendency of people to use a 50% response to indicate uncertainty rather than a true judgement of probability (18). We also saw that about a quarter of respondents on the first question stated their risk

For peer review only - http://bmjopen.bmj.com/site/about/guidelines.xhtml

was “average” or “higher”. This may point to considerable uncertainty in the public as to how to interpret test results. Use of the top end of the scale is hard to interpret but may either reflect a failure to read the information carefully and therefore a misunderstanding of the meaning of the result, or participants’ using information beyond the experiment to assess their risk and not adequately considering the hypothetical test result when making their response. Only 7% of the sample responded that their risk would be higher on the primary outcome measure. Reasons for this are unclear.

While we found no evidence for a direct effect on protective behaviours of the terms used to describe antibody tests results, there was indirect evidence that perceiving no risk of future infection might reduce frequency of handwashing. This finding is tentative, given it is based on behavioural intentions in response to a hypothetical antibody-positive result. Nonetheless the potential for antibody testing to increase viral transmission must be considered alongside the potential benefits the tests might have in allowing the easing of lockdown restrictions. Clear communication about the ongoing need for handwashing, in particular, will be essential and raising public awareness of the main mechanisms through which SARS-CoV-2 is transmitted – through air and surfaces – might help improve adherence. This, in addition to acknowledgment of the imperfect nature of the tests, will give the public a more accurate representation of the meaning and implications of an antibody test result and a better understanding of how to reduce the risk of transmission. Such communications need to emphasise that transmission can occur through contact regardless of antibody status. Such communications also need to be rigorously evaluated to ensure their effectiveness at communicating these points both to those undergoing antibody tests as well as to general populations that are now having to learn to live with SARS-CoV-2.

It was notable that 85% of respondents said that they would probably or definitely have an antibody test if they were offered it. This suggests high interest but should be treated with caution not least because fewer undergo than express interest in undergoing tests (19).

For peer review only

Strengths and Limitations

This study provides the first experimental evidence for the potentially adverse impact on risk perceptions and protective behaviours of commonly used terms to describe SARS-CoV-2 antibody tests and their results. As such, it provides timely evidence to inform policy and research to mitigate these effects to realise the potential benefits of such tests.

The study has several limitations. First, participants were responding to a hypothetical test and asked to imagine that they had received a test result that had detected antibodies. The extent to which people responded using the information provided in the study is unknown. Some will likely have used other information about SARS-CoV-2 and their own experiences to answer the questions about risk. This is a limitation of all hypothetical scenario-based studies of this type but findings from such studies can generalise to clinical settings (20,21) although some caution is warranted. As antibody testing is not yet routinely being carried out in the UK, the impact of the wording used in the current study amongst those undergoing testing cannot be assessed. All scenarios used the term 'positive' so we were unable to examine any possible impact of the use of this term. In addition, due to the rapidity with which the current study was conducted, piloting of the materials in general population samples was not carried out. It is therefore possible that some of the information provided may have been misinterpreted.

Second, the protective behaviours of handwashing and physical distancing were measured using single items assessing behavioural intentions following a hypothetical test result.

Third, the sample size was insufficient to detect effect sizes that could be important at a population level. It is possible, for example, that the use of the terms Certificate or Passport might impact on risk perception, but the current study lacked the power to detect this.

Fourth, while quotas were used to achieve a sample broadly representative of the UK population, research panels are not representative of the general population (22,23). We found no evidence that the impact of the interventions in this study was modified by demographic characteristics of the participants, providing some reassurance about the generalisability of results across age groups, gender, educational level and geographical region of the UK.

Implications for research and policy

The results of this study have several implications for research and policy. The effectiveness of antibody tests for SARS-CoV-2 will depend not only on the extent and duration of any immunity conferred and the performance of a test, but also upon a good understanding of the meaning of tests results among those offered them. First, the use of the term Immunity should be avoided in phrases to describe antibody tests, whether described as Passports, Certificates or Tests. This has implications for the presentation of antibody testing by policy-makers and the media,

For peer review only - http://bmjopen.bmj.com/site/about/guidelines.xhtml

as well as those considering the wording of test result letters sent to tested individuals. Second, research is needed to evaluate different ways of informing those offered tests and receiving tests results to minimise the proportion erroneously perceiving an antibody-positive test result to mean no risk of becoming infected with the virus. It should also focus on maximising understanding that – regardless of antibody-status – anyone can indirectly transmit the virus by touching a contaminated surface and infecting the next surface they touch. Hand washing or sanitising therefore need to remain frequent. Research is also needed with those undergoing actual tests, powered to detect effects judged meaningful in the context of a population-based testing programme and involving measures of actual behaviour.

Conclusion

Interest in SARS-CoV-2 antibody testing is high – across many countries, employers and populations. While such testing could contribute to wider strategies to ease lock-down restrictions, their use may have an adverse impact on transmission-related behaviour. This appears to vary with the way the tests are described. Using the term Immunity (vs Antibody) to describe antibody tests increases the proportion of people believing that an antibody-positive result means they have no future risk of coronavirus, a perception that may be associated with less frequent handwashing and hence increased risk of transmission.

For peer review only

Figure 1 **Proportion believing an antibody-positive test result means 'no risk' of future infection** *Error bars show 95% confidence intervals*

See separate file for Figure 1

Figure 2 **Perceived meaning of an antibody-positive test result for future risk and intentions to reduce frequency of hand washing** *Error bars show 95% confidence intervals*

See separate file for Figure 2

For peer review only

For peer review only - http://bmjopen.bmj.com/site/about/guidelines.xhtml

References

1. Hale T, Webster S, Petherick A, Phillips T, Kira B. *Oxford COVID-19 Government Response Tracker*, Blavatnik School of Government [Internet]. 2020. Available from: https://www.bsg.ox.ac.uk/research/research-projects/coronavirus-government-response-tracker

2. World Health Organization. *Critical preparedness, readiness and response actions for COVID-19* [Internet]. World Health Organization; 2020. (Interim Guidance 22 March 2020). Report No.: WHO/2019-nCoV/Community_Actions/2020.3. Available from: https://www.who.int/publications-detail/critical-preparedness-readiness-and-response-actions-for-covid-19

3. Studdert DM, Hall MA. Disease Control, Civil Liberties, and Mass Testing — Calibrating Restrictions during the Covid-19 Pandemic. *N Engl J Med* [Internet]. 2020 Apr 9; Available from: https://doi.org/10.1056/NEJMp2007637

4. Proctor K, Sample I, Oltermann P. 'Immunity passports' could speed up return to work after Covid-19. *The Guardian* [Internet]. 2020 Mar 30; Available from: https://www.theguardian.com/world/2020/mar/30/immunity-passports-could-speed-up-return-to-work-after-covid-19

5. Slotnick D. Delta's CEO said he would support an 'immunity passport' program or other steps to jumpstart travel as the airline reports its first quarterly loss in more than 5 years. *Business Insider* [Internet]. Available from: https://www.businessinsider.com/delta-first-quarter-coronavirus-air-travel-outlook-after-the-pandemic-2020-4

6. Merrick R. People could be given coronavirus 'immunity certificates' to leave lockdown early. *The Independent* [Internet]. 2020 Apr 2; Available from: https://www.independent.co.uk/news/uk/politics/coronavirus-uk-immunity-certificate-boris-johnson-test-germany-a9442211.html

7. Giugliano F. Mass Coronavirus Antibody Tests Have Serious Limits. *Bloomberg.com* [Internet]. 2020 Apr 24; Available from: https://www.bloomberg.com/opinion/articles/2020-04-24/coronavirus-mass-covid-19-antibody-tests-have-serious-limits

8. Forgey Q. Fauci: Coronavirus immunity cards for Americans are 'being discussed'. *POLITICO* [Internet]. Available from: https://www.politico.com/news/2020/04/10/fauci-coronavirus-immunity-cards-for-americans-are-being-discussed-178784

9. Sherwood D. Chile to push ahead with coronavirus 'release certificates' despite WHO warning. *Reuters* [Internet]. 2020 Apr 26; Available from: https://www.reuters.com/article/us-health-coronavirus-chile-idUSKCN2280NW

10. Adams ER, Anand R, Andersson MI, Auckland K, Baillie JK, Barnes E, et al. Evaluation of antibody testing for SARS-Cov-2 using ELISA and lateral flow immunoassays. *MedRxiv* Prepr. 2020 Jan 1;2020.04.15.20066407.

For peer review only - http://bmjopen.bmj.com/site/about/guidelines.xhtml

11. Altmann DM, Douek DC, Boyton RJ. What policy makers need to know about COVID-19 protective immunity. *The Lancet* [Internet]. 2020 Apr 27. Available from: https://www.thelancet.com/journals/lancet/article/PIIS0140-6736(20)30985-5/abstract

12. Loong T-W. Understanding sensitivity and specificity with the right side of the brain. *BMJ*. 2003;327(7417):716–719.

13. Marteau T, Saidi G, Goodburn S, Lawton J, Michie S, Bobrow M. Numbers or words? A randomized controlled trial of presenting screen negative results to pregnant women. Published in Affiliation with the International Society for Prenatal Diagnosis. *Prenat Diagn* 2000209714-718.

14. Marteau TM, Senior V, Sasieni P. Women's understanding of a "normal smear test result": experimental questionnaire based study. *BMJ*. 2001;322(7285):526–528.

15. World Health Organization. *'Immunity passports' in the context of COVID-19* [Internet]. World Health Organization. Report No.: WHO/2019-nCoV/Sci_Brief/Immunity_passport/2020.1. Available from: https://www.who.int/publications-detail/immunity-passports-in-the-context-of-covid-19

16. Jaki T, Vasileiou D. Factorial versus multi-arm multi-stage designs for clinical trials with multiple treatments. *Stat Med*. 2017 Feb 20;36(4):563–80.

17. Lohm D, Davis M, Flowers P, Stephenson N. 'Fuzzy' virus: indeterminate influenza biology, diagnosis and surveillance in the risk ontologies of the general public in time of pandemics. *Health Risk Soc*. 2015 Feb 17;17(2):115–31.

18. de Bruin WB, Fischhoff B, Millstein SG, Halpern-Felsher BL. Verbal and Numerical Expressions of Probability: 'It's a Fifty-Fifty Chance'. *Organ Behav Hum Decis Process*. 2000 Jan;81(1):115–31.

19. Cooke R, French DP. How well do the theory of reasoned action and theory of planned behaviour predict intentions and attendance at screening programmes? A meta-analysis. *Psychol Health*. 2008 Oct 1;23(7):745–65.

20. Wright AJ, Takeichi C, Whitwell SCL, Hankins M, Marteau TM. The impact of genetic testing for Crohn's disease, risk magnitude and graphical format on motivation to stop smoking: an experimental analogue study. *Clin Genet*. 2008 Apr;73(4):306–14.

21. Hollands GJ, Whitwell SCL, Parker RA, Prescott NJ, Forbes A, Sanderson J, et al. Effect of communicating DNA based risk assessments for Crohn's disease on smoking cessation: randomised controlled trial. *BMJ* [Internet]. 2012 Jul 20;345. Available from: https://www.bmj.com/content/345/bmj.e4708

22. ONS. Internet users, UK - Office for National Statistics [Internet]. Office for National Statistics; 2019 May. Available from: https://www.ons.gov.uk/businessindustryandtrade/itandinternetindustry/bulletins/internetusers/2019

23. Wright KB. Researching Internet-Based Populations: Advantages and Disadvantages of Online Survey Research, Online Questionnaire Authoring Software Packages, and Web Survey Services. *J Comput-Mediat Commun*. 2005;10(3).

For peer review only - http://bmjopen.bmj.com/site/about/guidelines.xhtml

Funding

Data collection for this study was funded by a block UK government grant to the Behavioural Insights Team. JW is funded by a career development fellowship from Cancer Research UK (ref C7492/A17219). GJR is funded by the National Institute for Health Research Health Protection Research Unit (NIHR HPRU) in Emergency Preparedness and Response at King's College London in partnership with Public Health England (PHE), in collaboration with the University of East Anglia. The views expressed in this paper are those of the authors and not necessarily those of UK government, Cancer Research UK, NIHR or Public Health England.

Competing interests

All authors have completed the Unified Competing Interest form (available on request from the corresponding author) and declare: no support from any organisation for the submitted work; and no financial relationships with any organisations that might have an interest in the submitted work in the previous three years. HWWP declares consultancy fees from Babylon Health; all authors declare no other relationships or activities that could appear to have influenced the submitted work.

Transparency declaration

The authors affirm that the manuscript is an honest, accurate, and transparent account of the study being reported; that no important aspects of the study have been omitted; and that any discrepancies from the study as originally planned have been explained.

Data sharing statement

Anonymised data will be made available upon reasonable request.

Author contribution statement

For peer review only

The study was conceptualised by TMM, JW & GJR. AM completed data collection. JW & HP analysed the data. All authors contributed to, and approved, the final manuscript.

Acknowledgements

We thank Steve Reicher for comments on an earlier draft of the study protocol.

For peer review only

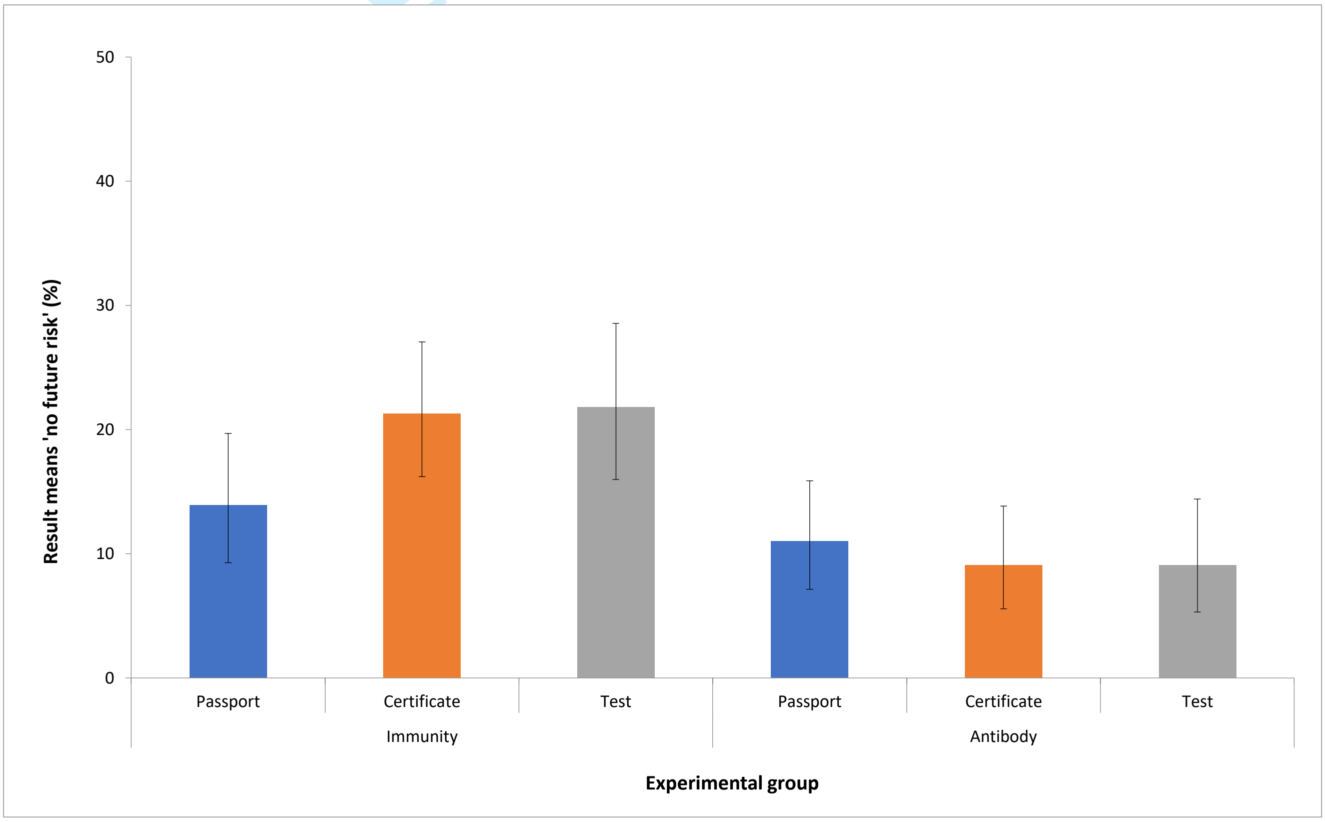

Result means 'no future risk' (%)
50
40
30
20
10
0
Passport
Certificate
Test
Passport
Certificate
Test
Immunity
Antibody
Experimental group

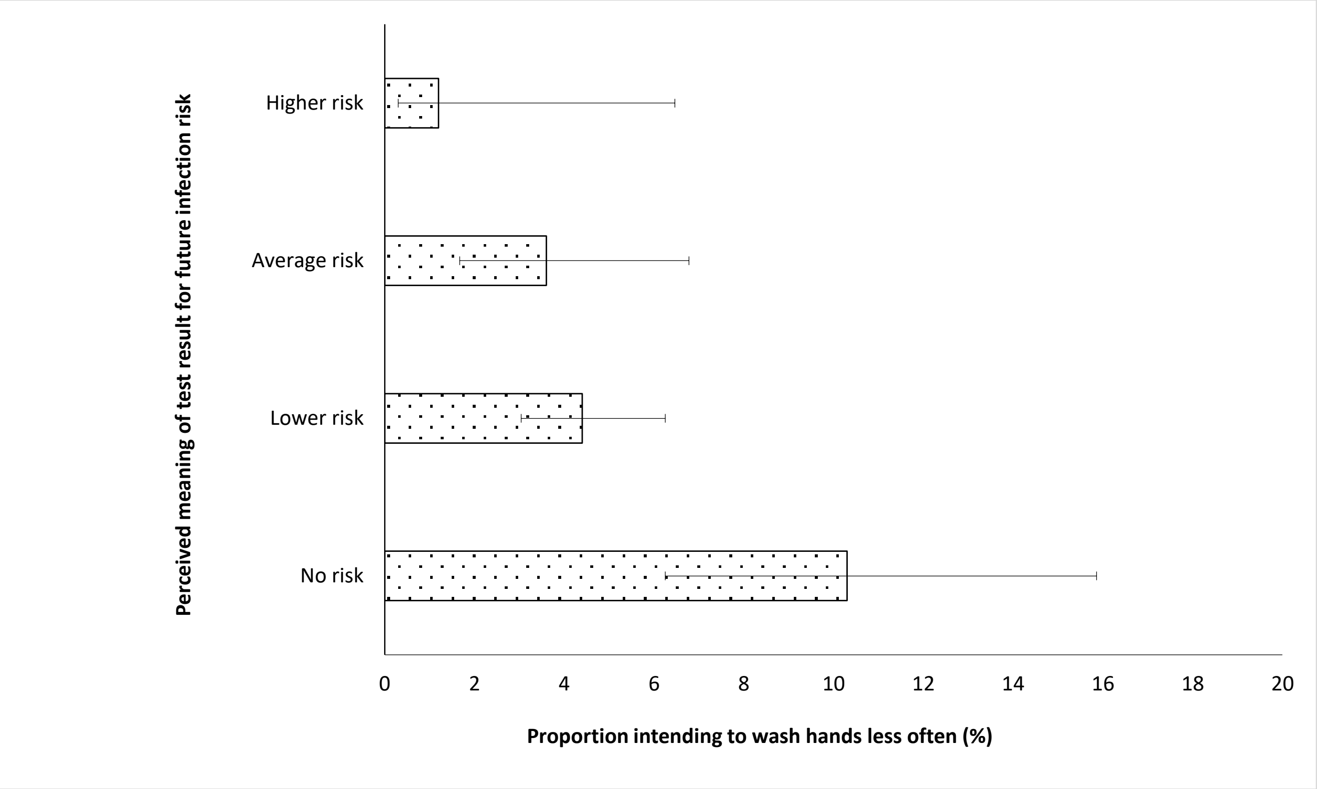
Perceived meaning of test result for future infection risk
Higher risk
Average risk
Lower risk
No risk
0
2
4
6
8
10
12
14
16
18
20
Proportion intending to wash hands less often (%)

Supplementary Materials

S1 Interventions

Intervention A

Immunity Passport

Scientists are developing tests to see who has already had coronavirus.

No test is 100% effective.

This means that those who test 'positive' would have:

- Lower risk of catching coronavirus in the future - and therefore also
- Lower risk of passing it on to others

Those who test 'positive' would get an immunity passport.

They could return to work early.

Intervention B

Immunity Certificate

Scientists are developing tests to see who has already had coronavirus.

No test is 100% effective.

This means that those who test 'positive' would have:

- Lower risk of catching coronavirus in the future - and therefore also
- Lower risk of passing it on to others

Those who test 'positive' would get an immunity certificate.

They could return to work early.

Intervention C

Immunity Test

Scientists are developing tests to see who has already had coronavirus.

No test is 100% effective.

This means that those who test ‘positive’ would have:

- Lower risk of catching coronavirus in the future - and therefore also
- Lower risk of passing it on to others

Those who test ‘positive’ would get a result showing immunity.

They could return to work early.

Intervention D

Antibody Passport

Scientists are developing tests to see who has already had coronavirus.

No test is 100% effective.

This means that those who test ‘positive’ would have:

- Lower risk of catching coronavirus in the future - and therefore also
- Lower risk of passing it on to others

Those who test ‘positive’ would get an antibody passport.

They could return to work early.

Intervention E

Antibody Certificate

Scientists are developing tests to see who has already had coronavirus.

No test is 100% effective.

This means that those who test ‘positive’ would have:

- Lower risk of catching coronavirus in the future - and therefore also
- Lower risk of passing it on to others

Those who test ‘positive’ would get an antibody certificate.

They could return to work early.

Intervention F

Antibody Test

Scientists are developing tests to see who has already had coronavirus.

No test is 100% effective.

This means that those who test 'positive' would have:

- Lower risk of catching coronavirus in the future - and therefore also
- Lower risk of passing it on to others

Those who test 'positive' would get a result showing antibodies.

They could return to work early.

For peer review only

S2. Measures

Primary outcome

I Imagine you were given *[an immunity passport/an immunity certificate/a result showing immunity/an antibody passport/an antibody certificate/a result showing antibodies]*

which one of the following statements is true*: [order of response options to be randomised]*

1. I have no risk of catching coronavirus in the future
2. I have a lower risk of catching coronavirus in the future
3. I have an average risk of catching coronavirus in the future
4. I have a higher risk of catching coronavirus in the future

Secondary outcomes

II Imagine you were given *[an immunity passport/an immunity certificate/a result showing immunity/an antibody passport/an antibody certificate/a result showing antibodies]*

how likely is it that you will get coronavirus at some point in the future?

Please answer on a scale from 0 % to 100% where 0% means no chance and 100% means certain.

0% ---100%

III Imagine that you were given *[an immunity passport/an immunity certificate/a result showing immunity/an antibody passport/an antibody certificate/a result showing antibodies]*

would you wash your hands with soap and water or sanitiser:

1. Much more than now
2. More than now
3. Same as now
4. Less than now
5. Much less than now

IV Imagine you were given *[an immunity passport/an immunity certificate/a result showing immunity/an antibody passport/an antibody certificate/a result showing antibodies]*

would you avoid physical contact with others outside of your home:

1. Much more than now
2. More than now
3. Same as now
4. Less than now
5. Much less than now

V If you were offered a test today by the NHS to check whether you have ever had coronavirus, would you have it?

1. Yes, definitely
2. Yes, probably
3. No, probably not
4. No, definitely not

For peer review only

Supplementary Table 1 – Logistic regression analysis examining impact of Test type and Result type on protective behaviours

	Intend to wash hands less			Intend to avoid contact less		
	% responding less/much less in each sub-group % (95% CI)	Mutually adjusted (n=1204)	Adjusted for demographics[1] (n=1179)	% responding less/much less in each sub-group % (95% CI)	Mutually adjusted (n=1204)	Adjusted for demographics[1] (n=1179)
Test Type						
Antibody (n=603)	4.1 (2.7-6.1)	Ref	Ref	17.2 (104)	Ref	Ref
Immunity (n=601)	5.7 (4.0-7.8)	1.38 (0.43-4.45)	1.62 (0.46-5.73)	22.1 (133)	1.40 (0.82-2.39)	1.46 (0.85-2.50)
Result Type						
Test (n=354)	3.4 (1.8-5.9)	Ref	Ref	19.2 (68)	Ref	Ref
Certificate (n=444)	6.8 (4.6-9.5)	2.07 (0.72-6.00)	2.47 (0.77-7.96)	21.6 (96)	1.16 (0.68-1.96)	1.14 (0.67-1.96)
Passport (n=406)	4.2 (2.5-6.6)	1.29 (0.41-4.01)	1.79 (0.52-6.16)	18.0 (73)	0.99 (0.58-1.69)	1.02 (0.59-1.76)
Test by Result interaction						
Certificate by Immunity result		0.98 (0.25-3.95)	0.80 (0.18-3.56)		1.00 (0.49-2.01)	0.94 (0.46-1.93)
Passport by Immunity result		0.96 (0.21-4.40)	0.58 (0.11-2.98)		0.89 (0.43-1.87)	0.84 (0.40-1.78)

[1]Fully adjusted model includes age (with quadratic term), gender, education level and region (coded as per Table 1)

For peer review only

Supplementary Table 2 – Logistic regression examining impact of Test and Result type on intention to have the test

	Do not intend to have the test		
	Proportion who would not have test in each sub-group % (95% CI)	Mutually adjusted (n=1204)	Adjusted for demographics[1] (n=1179)
Test Type			
Antibody (n=603)	13.1 (10.5-16.1)	Ref	Ref
Immunity (n=601)	16.5 (13.6-19.7)	1.02 (0.57-1.81)	0.97 (0.54-1.74)
Result Type			
Test (n=354)	15.5 (11.9-19.7)	Ref	Ref
Certificate (n=444)	14.9 (11.7-18.5)	0.71 (0.39-1.28)	0.63 (0.34-1.15)
Passport (n=406)	14.0 (10.8-17.8)	0.80 (0.54-1.42)	0.82 (0.46-1.47)
Test by Result interaction			
Certificate by Immunity result		1.65 (0.75-3.63)	1.91 (0.84-4.31)
Passport by Immunity result		1.23 (0.55-2.75)	1.22 (0.54-2.78)

[1]Fully adjusted model includes age (with quadratic term), gender, education level and region (coded as per Table 1)

For peer review only

Supplementary Table 3 – Logistic regression analysis examining impact of perceived risk on anticipated behaviour

	Intend to wash hands less			Intend to avoid contact less		
	% responding less/much less in each sub-group % (95% CI)	Unadjusted (n=1204)	Adjusted for demographics[1] (n=1179)	% responding less/much less in each sub-group % (95% CI)	Unadjusted (n=1204)	Adjusted for demographics[1] (n=1179)
Perceived meaning of result						
Some residual risk (n=1030)	4.0 (2.8-5.3)	Ref	Ref	18.8 (16.5-21.4)	Ref	Ref
No residual risk of future infection (n=175)	10.3 (6.3-15.9)	**2.78 (1.56-4.97)**	**2.32 (1.25-4.28)**	24.7 (18.5-31.8)	1.41 (0.97-2.06)	1.37 (0.93-2.03)

[1]Fully adjusted model includes age (with quadratic term), gender, education level and region (coded as per Table 1)

For peer review only

"Immunity Passports" for SARS-CoV-2: an online experimental study of the impact of antibody test terminology on perceived risk and behaviour

Journal:	*BMJ Open*
Manuscript ID	bmjopen-2020-040448.R2
Article Type:	Original research
Date Submitted by the Author:	22-Jul-2020
Complete List of Authors:	Waller, Jo; King's College London, School of Cancer and Pharmaceutical Sciences<br>Rubin, GJ; King\'s College London School of Medical Education<br>Potts, Henry; UCL, Centre for Health Informatics and Multiprofessional Education<br>Mottershaw, Abigail; Behavioural Insights Team<br>Marteau, Theresa; University of Cambridge, Behaviour and Health Research Unit
**Primary Subject Heading**:	Public health
Secondary Subject Heading:	Infectious diseases
Keywords:	Public health < INFECTIOUS DISEASES, PUBLIC HEALTH, IMMUNOLOGY

SCHOLARONE™
Manuscripts

I, the Submitting Author has the right to grant and does grant on behalf of all authors of the Work (as defined in the below author licence), an exclusive licence and/or a non-exclusive licence for contributions from authors who are: i) UK Crown employees; ii) where BMJ has agreed a CC-BY licence shall apply, and/or iii) in accordance with the terms applicable for US Federal Government officers or employees acting as part of their official duties; on a worldwide, perpetual, irrevocable, royalty-free basis to BMJ Publishing Group Ltd ("BMJ") its licensees and where the relevant Journal is co-owned by BMJ to the co-owners of the Journal, to publish the Work in this journal and any other BMJ products and to exploit all rights, as set out in our licence.

The Submitting Author accepts and understands that any supply made under these terms is made by BMJ to the Submitting Author unless you are acting as an employee on behalf of your employer or a postgraduate student of an affiliated institution which is paying any applicable article publishing charge ("APC") for Open Access articles. Where the Submitting Author wishes to make the Work available on an Open Access basis (and intends to pay the relevant APC), the terms of reuse of such Open Access shall be governed by a Creative Commons licence – details of these licences and which Creative Commons licence will apply to this Work are set out in our licence referred to above.

Other than as permitted in any relevant BMJ Author's Self Archiving Policies, I confirm this Work has not been accepted for publication elsewhere, is not being considered for publication elsewhere and does not duplicate material already published. I confirm all authors consent to publication of this Work and authorise the granting of this licence.

For peer review only

WALLER et al FINAL BMJ Open R2 MS 20 July 2020

"Immunity Passports" for SARS-CoV-2: an online experimental study of the impact of antibody test terminology on perceived risk and behaviour

Jo Waller PhD[1], G. James Rubin PhD[2], Henry W. W. Potts PhD[3], Abi Mottershaw[4], Theresa M Marteau PhD[5] *

[1] Jo Waller, Reader in Cancer Behavioural Science, School of Cancer and Pharmaceutical Sciences, King's College London, Guy's Hospital, Great Maze Pond, London, SE1 9RT.

[2] G. James Rubin, Reader in the Psychology of Emerging Health Risks. Department of Psychological Medicine, King's College London, Weston Education Centre, Cutcombe Road, London, SE5 9RJ.

[3] Henry W. W. Potts, Associate Professor, Institute of Health Informatics, University College London, 222 Euston Road London, NW1 2DA.

[4]Abigail L. Mottershaw, Online Experiments Lead. Behavioural Insights Team, 4 Matthew Parker Street, London, SW1H 9NP.

[*5] Theresa M Marteau, Director of Research, Behaviour and Health Research Unit, University of Cambridge, Institute of Public Health, Forvie Site, Robinson Way, Cambridge, CB2 0SR.

* corresponding author: tm388@cam.ac.uk

For peer review only - http://bmjopen.bmj.com/site/about/guidelines.xhtml

WALLER et al FINAL BMJ Open R2 MS 20 July 2020

Objective: To assess the impact of describing an antibody-positive test result using the terms Immunity and Passport or Certificate, alone or in combination, on perceived risk of becoming infected with SARS-CoV-2 and protective behaviours.

Design: 2 × 3 experimental design.

Setting: Online

Participants: 1,204 adults from a UK research panel.

Intervention: Participants were randomised to receive one of six descriptions of an antibody test and results showing SARS-CoV-2 antibodies, differing in the terms describing the type of test (Immunity *vs* Antibody) and the test result (Passport *vs* Certificate *vs* Test).

Main outcome measures: Primary outcome: proportion of participants perceiving no risk of infection with SARS-CoV-2 given an antibody positive test result. Other outcomes include: intended changes to frequency of handwashing and physical distancing.

Results: When using the term Immunity (vs Antibody), 19.1% of participants [95% CI: 16.1 to 22.5] (vs 9.8% [95% CI: 7.5 to 12.4]) perceived no risk of catching coronavirus given an antibody-positive test result (AOR: 2.91 [95% CI: 1.52 to 5.55]). Using the terms Passport or Certificate – as opposed to Test – had no significant effect (AOR: 1.24 [95% CI: 0.62 to 2.48] and AOR: 0.96 [95% CI: 0.47 to 1.99] respectively). There was no significant interaction between the effects of the test and result terminology. Across groups, perceiving no risk of infection was associated with an intention to wash hands less frequently (AOR: 2.32 [95% CI: 1.25 to 4.28]); there

For peer review only - http://bmjopen.bmj.com/site/about/guidelines.xhtml

was no significant association with intended avoidance of physical distancing (AOR: 1.37 [95% CI: 0.93 to 2.03]).

Conclusions: Using the term Immunity (vs Antibody) to describe antibody tests for SARS-CoV-2 increases the proportion of people believing that an antibody-positive result means they have no risk of catching coronavirus in the future, a perception that may be associated with less frequent handwashing.

Key words: SARS-CoV-2; COVID-19; antibody testing; immunity; passport; certificate; risk perception; protective behaviours

Study registration: Open Science Framework: https://osf.io/tjwz8/files/

For peer review only

Strengths and limitations of this study

- This study provides the first experimental evidence for the potentially adverse impact on risk perceptions and protective behaviours of using the term Immunity – as opposed to Antibody - to describe SARS-CoV-2 antibody tests and their results.
- It provides timely evidence to inform policy and research to mitigate these effects to realise the potential benefits of such tests.
- Participants were responding to a hypothetical test and asked to imagine that they had received a test result that had detected antibodies.
- Protective behaviours of handwashing and physical distancing were measured using single items assessing behavioural intentions.

For peer review only

For peer review only - http://bmjopen.bmj.com/site/about/guidelines.xhtml

Introduction

At the height of the first wave of the COVID-19 pandemic, about a third of the world's population is estimated to have been in lockdown, with all but essential workers largely confined to home (1).

Without an effective treatment or vaccine, testing for infection combined with contact tracing and isolation will be central to effective strategies to ease populations out of lockdown while keeping the basic reproduction number (R_0) below one (2).

Testing for antibodies to SARS-CoV-2 is a possible complement to testing for active infection to identify those who have developed antibodies to the virus and so may be able to return to work and other activities without significantly increasing transmission rates (3). These tests have been variously described in the media as Immunity Passports (4,5), Immunity Certificates (6,7) Immunity Cards (8) and Release Certificates (9). Unfortunately, the use of these terms implies a certainty unmatched by current evidence about antibody tests (10). But whether these terms actually encourage a misplaced sense of certainty even before testing is widely available is unknown and the focus of the current study.

Uncertainties inherent in tests for antibodies to SARS-CoV-2 include the extent and duration of immunity conferred (11). They also include the uncertainties inherent in any test regarding the proportion of those who would be correctly identified. This depends upon the test performance – its sensitivity and specificity – as well as the population prevalence of the tested condition (12). Given these uncertainties, those who receive a test result indicating the presence of antibodies will have a residual risk of becoming infected by SARS-CoV-2 in the future.

For peer review only - http://bmjopen.bmj.com/site/about/guidelines.xhtml

Understanding that there is this residual risk – albeit one that is difficult to quantify at present – will be important to minimise transmission that could arise from those receiving "antibody positive" test results. If people testing positive perceive that they have no risk of becoming infected by the virus, they may ignore any future symptoms of infection and facilitate transmission if they fail to self-isolate appropriately. Such a perception may also over-generalise to a belief that they are unable to transmit infection through contact with contaminated surfaces. Regardless of antibody status, all individuals can indirectly transmit the virus between surfaces by touch. Hand washing or sanitising therefore need to remain frequent.

Evidence from other testing programmes suggests that interpreting a low risk result to mean no risk can be reduced by verbal and numerical expressions of residual risk when presenting test results (13,14). But even before testing programmes are in place, the terms commonly used to describe these tests – Immunity Passport or Certificates – may inadvertently be fuelling a misplaced sense of certainty about their results. It is unknown whether describing these tests as being for immunity – as opposed to antibodies – or their results as passports or certificates increases misunderstanding of the residual risk inherent in an antibody-positive test result and thereby reducing adherence to protective behaviours and increasing risk of transmission (15).

This study was designed to test two hypotheses: describing a test indicating the presence of antibodies using the term Immunity (vs Antibody), and describing test results as Passports or Certificates (vs Test), increases the likelihood that those with this test result erroneously perceive they have no risk of becoming infected in the future with coronavirus.

Methods

For peer review only

Ethical approval for this study was granted by the King's College London Research Ethics Committee (reference: MRA-19/20-18685).

The protocol was preregistered on the Open Science Framework https://osf.io/tjwz8/ *Study 2*

The statistical analysis plan was pre-specified and uploaded to the Open Science Framework prior to receipt of the data https://osf.io/tjwz8/ *Study 2*

An initial study with similar methods was conducted https://osf.io/tjwz8/ *Study 1* but, due to an error, the intervention was not correctly programmed. This study is therefore not reported.

Design

The study was an online experiment using a 2 × 3 factorial design, with participants randomised, with an equal allocation ratio, to one of six groups varying in the description of an antibody test and a result showing the presence of antibodies. These descriptions differed only in the term used for what was being tested (Immunity *vs* Antibody) and the term used for the test result (Passport *vs* Certificate *vs* Test).

Participants

A quota sample of 1,204 adults was recruited via Predictiv, the Behavioural Insights Team's online experimentation platform (https://www.bi.team/bi-ventures/predictiv/) comprising 500,000 adults in the UK. Quotas were based on age, gender and UK region to achieve a sample broadly representative of the UK population. 1373 clicked on the link to enter the study of whom 1214 subsequently completed the study. Ten were excluded for failing to meet quality checks (e.g. the unique participant identifier

For peer review only - http://bmjopen.bmj.com/site/about/guidelines.xhtml

was missing, or the participant had entered the survey more than once). Participants were reimbursed in points (equivalent to £1) which could be redeemed in cash, gift vouchers or charitable donations. Participants did not know the topic of the study prior to participation. All participants had provided opt-in consent to take part in surveys when they signed up to the online panel. No identifiable data were collected as part of the survey. All data collected by Predictiv are processed in line with their published privacy policy (http://www.predictiv.co.uk/privacy-policy.html).

Patient and public involvement

Due to the rapid nature of this research, the public was not involved in the development of the study.

Power

The sample size was chosen pragmatically without reference to a specific power calculation. We fitted a full linear model with two levels for test type (immunity/antibody), three levels for result type (passport/certificate/test) and an interaction term. Conservatively, we then had an 80% chance of detecting, at a 5% significance level, an increase in the primary outcome measure from 50% in a baseline group to 64% in another group. The power calculation was done using http://powerandsamplesize.com/, taking 50% as the baseline to provide a conservative estimate of power.

Randomisation

Participants were randomised to groups by random number generation. A random number between 1 and 6 was generated for every participant upon entry to the study to determine which description they saw, with each of the six numbers corresponding to one of the six description. As this is based on true randomness, the number of participants within each group can vary due to chance (see Table 1 for numbers in each group).

For peer review only - http://bmjopen.bmj.com/site/about/guidelines.xhtml

Intervention

The intervention comprised a description of antibody testing and test results indicating the presence of antibodies [see Box 1 for one example and S1 for wording of all six descriptions]. These differed across six groups in test name of results indicating the presence of antibodies. All descriptions included the information that the result would mean a lower risk of future infection and transmission, and that people with this result could return to work earlier.

Box Immunity Passport: one of the six descriptions of an antibody-positive test result

Immunity Passport

Scientists are developing tests to see who has already had coronavirus.

No test is 100% effective.

This means that those who test 'positive' would have:

- Lower risk of catching coronavirus in the future, and therefore also
- Lower risk of passing it on to others

Those who test 'positive' would get an immunity passport.

They could return to work early.

Outcomes measures

Wording of the items used for each measure is shown in Supplementary Materials (S2). Response options to all outcome measures are shown in Table 2.

Primary outcome

Proportion of participants perceiving an antibody-positive test result to mean no risk of catching coronavirus in the future, assessed in response to a question with four response options.

For peer review only - http://bmjopen.bmj.com/site/about/guidelines.xhtml

Secondary outcomes

Perceived likelihood of catching coronavirus in the future, assessed on a visual analogue scale from 0% to 100%.

Intention to engage in handwashing less or more frequently than now, given an antibody-positive test result: assessed in response to a question with five response options.

Intention to avoid physical contact with others outside the home more or less frequently than now, given an antibody-positive test result: assessed in response to a question with five response options.

Interest in undergoing the test if offered today: assessed in response to a question with four response options.

Other measures

Demographic characteristics: age, gender, level of education and geographical region of residence. Employment status, planned to be included, was omitted due to a technical error.

Statistical analyses

A detailed statistical analysis plan is available on the Open Science Framework, specified prior to receipt of the data https://osf.io/tjwz8/ *Study 2*. Binary logistic regression was used to assess the impact of test type (immunity/antibody) and result type (passport/certificate/test) on the odds of believing the antibody test result means there is no risk of future infection. An interaction term was included in the model (16). The analysis was repeated adjusting for age (including a quadratic function to model

For peer review only

a non-linear relationship), gender, education and region based on prior results showing these are predictors of risk beliefs.

Binary logistic regressions were run (as above) for the secondary outcomes: intention to wash hands less, intention to engage less in social distancing and intention to undergo the test. Unadjusted and adjusted odds ratios and 95% confidence intervals are reported. Logistic regression was run to assess the extent to which intentions to engage in less frequent handwashing or social distancing measures is predicted by perceiving the test result to mean no risk of being infected in the future by coronavirus.

As only a very small proportion of participants gave a 'zero' response on the sliding scale of future risk, we used a linear regression model to examine this outcome, rather than a binary (zero vs. other) logistic regression as pre-specified in the analysis plan.

Procedure

Data were collected using an online survey platform, Predictiv. Upon entry to the study, participants were informed that they were to be asked some questions about coronavirus and that it would take about five minutes to complete. Participants were then shown one of six brief descriptions of an antibody test for coronavirus (see S1 for full text for each of the six descriptions). They were then asked five questions, assessing the primary and secondary outcomes. Participants' demographic characteristics were accessed from the survey platform.

Results

Sample characteristics

For peer review only - http://bmjopen.bmj.com/site/about/guidelines.xhtml

The sample comprised 606 women and 598 men with a median age of 36 years. Around a quarter had some graduate-level education (24.2%) and there was good representation of all UK regions (see Table 1). Distribution of sample characteristics by exposure group is shown in Table 1.

Descriptive statistics

Primary outcome

Responses to the five outcome questions for the whole sample and by experimental group are shown in Table 2. Overall, 14.5% of respondents [95% CI: 12.5 to 16.6] interpreted the test result as meaning they had no risk of future infection. Over half (57.8% [95% CI: 55.0 to 60.1]) interpreted the test result as meaning that their future risk of coronavirus was 'lower', in line with the description they had been given.

Secondary outcomes

Perceived level of future risk (on a scale of 0 to 100%) showed a complex, trimodal distribution. The median was 35% with an interquartile range from 18% to 51%. Only 5% of respondents put their risk at 0%. Overall, 63% put their risk below 50%. 10% put their risk at 50%, which was the modal response. 24% put their risk at greater than 50%, but below 100%. 3% of respondents put their risk at 100%: that is, they said they were certain to contract the virus. There was a lack of consistency between responses to the categorical and the continuous measures of risk. Median responses on the sliding scale were 12.5%, 28.0%, 50.0% and 65.0% in the 'no risk', 'lower risk', 'average risk' and 'higher risk' categories respectively, with responses ranging from 0-100 for all four of these groups. Although this suggests that a proportion of people were responding very differently to the two questions (e.g. 'no risk' on the first outcome and 100% risk on the sliding scale), the increasing median responses with increasing risk category provides some reassurance that most people's responses were broadly consistent across the two measures.

For peer review only - http://bmjopen.bmj.com/site/about/guidelines.xhtml

On the behavioural outcomes, 4.9% [95% CI: 3.8 to 6.3] said they would wash their hands less frequently than now if they received a positive result while 19.7% [95% CI: 17.5 to 22.1] said they would be less inclined to avoid physical contact with others outside the home. Intentions to have the test if offered were high, with 56.1% [95% CI: 53.2 to 58.9] saying they would definitely, and 29.2% [95% CI: 26.6 to 31.8] saying they would probably have the test if offered today.

Between-group differences in the primary outcome

When Test type (Immunity *vs.* Antibody), Result type (Certificate *vs.* Passport *vs.* Test) and an interaction term were entered into a logistic regression model predicting the belief that the test result meant 'no risk' of future infection (see Table 3), there was a significant effect of Test type, which persisted when we adjusted for demographic factors (age (including a quadratic term), gender, education level and UK region; AOR: 2.91 [95% CI: 1.52 to 5.55]. Those in the 'Immunity' group were more likely to believe the result meant 'no risk' than those in the Antibody group (19.1% [95% CI: 16.1 to 22.5] *vs.* 9.8% [95% CI: 7.5 to 12.4]) [Figure 1]. There was no significant effect of result type and no significant interaction.

Between-group differences in secondary outcomes

We analysed the continuous measures of future perceived risk of infection using a linear model (ANOVA) with two levels for test type, three levels for result type, and an interaction term. Overall, there was no significant effect: $F_{5,1198} = 1.46$, $p = 0.20$, adjusted $R^2 < 1\%$. We repeated the analysis adjusting for demographic factors as covariates. Overall, there was a significant effect: $F_{13,1165} = 1.88$, $p = 0.03$, adjusted $R^2 = 1\%$. This was because of a significant effect of age: as age increased, perceived risk decreased. There remained no significant effect of the experimental variables.

Logistic regression analyses examining the impact of Test type, Result type and their interaction on intentions to wash hands and avoid physical contact less frequently and on willingness to have the test are shown in Supplementary tables 1 and 2.

For peer review only - http://bmjopen.bmj.com/site/about/guidelines.xhtml

Neither Test type, Result type nor their interaction were significantly associated with these behavioural outcomes.

For peer review only

For peer review only - http://bmjopen.bmj.com/site/about/guidelines.xhtml

Table 1 – Demographic characteristics of participants by experimental group (n=1204)

Characteristics	All (n=1204)	Immunity Passport (n=187)	Immunity Certificate (n=235)	Immunity Test (n=179)	Antibody Passport (n=219)	Antibody Certificate (n=209)	Antibody Test (n=175)
Gender [n (%)]							
Female	606 (50.3)	86 (46.0)	126 (53.6)	94 (52.5)	112 (51.1)	98 (46.9)	90 (51.4)
Male	598 (49.7)	101 (54.0)	109 (46.4)	85 (47.5)	107 (48.9)	111 (53.1)	85 (48.6)
Age [median (interquartile range)]	36 (32)	35 (34)	36 (34)	36 (31)	38 (32)	34 (27)	36 (31)
Education [n (%)]							
Below degree	888 (73.8)	139 (74.3)	177 (75.3)	126 (70.4)	165 (75.3)	150 (71.8)	131 (74.9)
Degree or above	291 (24.2)	45 (24.1)	52 (22.1)	49 (27.4)	49 (22.4)	55 (26.3)	41 (23.4)
Missing	25 (2.1)	3 (1.6)	6 (2.6)	4 (2.2)	5 (2.3)	4 (1.9)	3 (1.7)
UK region [n (%)]							
England – London	160 (13.3)	24 (12.8)	33 (14.0)	27 (15.1)	25 (11.4)	27 (12.9)	24 (13.7)
England – Midlands	189 (15.7)	38 (20.3)	34 (14.5)	24 (13.4)	30 (13.7)	32 (15.3)	31 (17.7)
England – South & East	373 (31.0)	48 (25.7)	74 (31.5)	51 (28.5)	75 (34.2)	74 (35.4)	51 (29.1)
England – North	308 (25.6)	46 (24.6)	65 (27.7)	50 (27.9)	59 (26.9)	43 (20.6)	45 (25.7)
Scotland/Wales/NI	174 (14.5)	31 (16.6)	29 (12.3)	27 (15.1)	30 (13.7)	33 (15.8)	24 (13.7)

For peer review only

For peer review only - http://bmjopen.bmj.com/site/about/guidelines.xhtml

WALLER et al FINAL BMJ Open R2 MS 20 July 2020

Table 2 – Primary and secondary outcomes by experimental group (n=1204)

	All (n=1204)	Immunity Passport (n=187)	Immunity Certificate (n=235)	Immunity Test (n=179)	Antibody Passport (n=219)	Antibody Certificate (n=209)	Antibody Test (n=175)
Perceived meaning of result for future risk [n (%)]							
No risk	174 (14.5)	26 (13.9)	50 (21.3)	39 (21.8)	24 (11.0)	19 (9.1)	16 (9.1)
Lower risk (in line with description)	697 (57.9)	106 (56.7)	127 (54.0)	92 (51.4)	126 (57.5)	134 (64.1)	112 (64.0)
Average risk	248 (20.6)	40 (21.4)	42 (17.9)	36 (20.1)	54 (24.7)	41 (19.6)	35 (20.0)
Higher risk	85 (7.1)	15 (8.0)	16 (6.8)	12 (6.7)	15 (6.8)	15 (7.2)	12 (6.9)
Perceived absolute risk (0-100) [Mean (SD)]	37.7 (25.2)	40.4 (26.5)	37.0 (25.4)	34.2 (27.1)	39.5 (23.6)	36.9 (25.2)	37.9 (23.1)
Perceived residual risk [n (%)]							
1-100%	1144 (95.0)	179 (95.7)	219 (93.2)	161 (89.9)	214 (97.7)	200 (95.7)	171 (97.7)
0%	60 (5.0)	8 (4.3)	16 (6.8)	18 (10.1)	5 (2.3)	9 (4.3)	4 (2.3)
Intention to wash hands [n (%)]							
Much less than now	13 (1.1)	3 (1.6)	7 (3.0)	0 (0.0)	0 (0.0)	2 (1.0)	1 (0.6)
Less than now	46 (3.8)	6 (3.2)	11 (4.7)	7 (3.9)	8 (3.7)	10 (4.8)	4 (2.3)
Same as now	800 (66.4)	121 (64.7)	159 (67.7)	127 (70.9)	138 (63.0)	143 (68.4)	112 (64.0)
More than now	161 (13.4)	23 (12.3)	26 (11.1)	18 (10.1)	34 (15.5)	29 (13.9)	31 (17.7)
Much more than now	184 (15.3)	34 (18.2)	32 (13.6)	27 (15.1)	39 (17.8)	25 (12.0)	27 (15.4)
Intention to avoid physical contact [n (%)]							
Much less than now	36 (3.0)	2 (1.1)	9 (3.8)	10 (5.6)	4 (1.8)	6 (2.9)	5 (2.9)
Less than now	201 (16.7)	35 (18.7)	48 (20.4)	29 (16.2)	32 (14.6)	33 (15.8)	24 (13.7)
Same as now	642 (53.3)	96 (51.3)	116 (49.4)	96 (53.6)	116 (53.0)	126 (60.3)	92 (52.6)
More than now	178 (14.8)	28 (15.0)	34 (14.5)	21 (11.7)	44 (20.1)	27 (12.9)	24 (13.7)
Much more than now	147 (12.2)	26 (13.9)	28 (11.9)	23 (12.8)	23 (10.5)	17 (8.1)	30 (17.1)
Would you have the test if offered? [n (%)]							
No, definitely not	38 (3.2)	5 (2.7)	14 (6.0)	5 (2.8)	5 (2.3)	4 (1.9)	5 (2.9)
No, probably not	140 (11.6)	24 (12.8)	28 (11.9)	23 (12.8)	23 (10.5)	20 (9.6)	22 (12.6)
Yes, probably	351 (29.2)	54 (28.9)	67 (28.5)	53 (29.6)	62 (28.3)	58 (27.8)	57 (32.6)
Yes, definitely	675 (56.1)	104 (55.6)	126 (53.6)	98 (54.7)	129 (58.9)	127 (60.8)	91 (52.0)

For peer review only

Table 3 – Logistic regression analysis examining impact of Test type and Result type on perception that the test result means 'no risk'

	Proportion answering 'no risk' in each sub-group % (95% CI) (n=1204)	Result means "No Risk" of future infection Odds ratio (95% confidence interval)	
		Mutually adjusted (n=1204)	Adjusted for demographics[1] (n=1179)
Test Type			
Antibody (n=603)	9.8 (7.5-12.4)	Ref	Ref
Immunity (n=601)	19.1 (16.1-22.5)	**2.77 (1.48-5.17)**	**2.91 (1.52-5.55)**
Result Type			
Test (n=354)	15.5 (11.9-19.7)	Ref	Ref
Certificate (n=444)	15.5 (12.3-19.3)	0.99 (0.50-2.00)	0.96 (0.47-1.99)
Passport (n=406)	12.3 (9.3-15.9)	1.22 (0.63-2.38)	1.24 (0.62-2.48)
Test by result interaction			
Certificate by Immunity result		0.98 (0.42-2.27)	1.00 (0.42-2.40)
Passport by Immunity result		0.47 (0.20-1.12)	0.46 (0.19-1.12)

[1]Fully adjusted model includes age (with quadratic term), gender, education level and region (coded as per Table 1)

For peer review only

Association between test result meaning 'no risk' and behavioural intentions

Logistic regression analyses were used to examine belief that the result meant 'no risk' as a predictor of intention to wash hands and avoid physical contact less frequently, given a positive result (see Figure 2 and Supplementary table 3). In analyses adjusting for demographic factors, those who believed there was no residual risk were at increased odds of intending to wash their hands less (AOR: 2.32 [95% CI: 1.25 to 4.28]). The association with intentions to physically distance outside the home was not significant (AOR: 1.37 [95% CI: 0.93 to 2.03]).

Discussion

Using the term Immunity – as opposed to Antibody – to describe antibody tests for SARS-CoV-2 doubled the proportion who erroneously perceived they would have no risk of becoming infected with the virus in the future if they were given an antibody-positive test result, from 9.8% for Antibody to 19.1% for Immunity (AOR: 2.91 [95% CI: 1.52 to 5.55]). Using the terms Passport, Certificate or Test to describe the results had no significant effect on risk perception (AOR: 0.96 [95% CI: 0.47-1.99] for Certificate and 1.24 [0.62-2.48] for Passport, compared with Test). The terms used to describe the test and results had no significant direct impact on intentions to engage in the protective behaviours of handwashing or physical distancing. However, across conditions, a greater proportion of those perceiving the result to mean no risk intended to wash their hands less often (10.3% [95% CI: 6.3 to 15.9]) compared with 4.0% who understood there was a residual risk (4.3% [95% CI: 2.8 to 5.3]).

These was no significant association with intended frequency of avoiding physical contact with others outside of the home. Interest in undergoing the test was high – with 85.2% saying they would probably or definitely have it if offered – and was unaffected by the terms used to describe the tests.

For peer review only - http://bmjopen.bmj.com/site/about/guidelines.xhtml

This study was designed to test two hypotheses, providing strong support for the first, that describing a test indicating the presence of antibodies using the term Immunity (vs Antibody) increases the likelihood that those with this test result erroneously perceive they have no risk of becoming infected in the future with coronavirus. This likely reflects a certainty about risk of future infection implicit in lay understandings of the term immunity that is not implied by the term antibody (17). Qualitative studies could explore this and other potential mechanisms for the effect observed.

The results of this study did not support the second hypothesis that describing test results as Passports or Certificates increases the likelihood that those with this test result erroneously perceive they have no risk of becoming infected in the future with coronavirus. This does not mean that these terms are unproblematic however, only that they did not influence the specific perceptions that we explored. Qualitative studies are warranted to understand the broader meanings these terms have in the context of testing for antibodies for SARS-CoV-2 and other contexts.

Responses on the sliding scale of future risk showed a high variability and were largely unexplained by the experimental intervention or other variables measured. They also showed only broad consistency with responses to the main outcome measure; some participants who believed the result meant 'no risk' then rated their future risk as 100%, and vice versa. This may point to considerable uncertainty in the public as to how to interpret test results and is consistent with the literature on risk communication and perception (18,19). Some participants may have been responding solely on the information provided; others may have been using other information about coronavirus and immunity to make judgements about their own risk. It also likely reflects the well-described tendency of people to use a 50% response to indicate uncertainty rather than a true judgement of probability (20). We

For peer review only - http://bmjopen.bmj.com/site/about/guidelines.xhtml

also saw that about a quarter of respondents on the first question stated their risk was "average" or "higher". This may point to considerable uncertainty in the public as to how to interpret test results. Use of the top end of the scale is hard to interpret but may either reflect participants' not reading the information carefully and therefore misunderstanding the meaning of the result being communicated in the information, or their using information beyond the experiment to assess their risk. Participants likely considered many factors in making sense of the hypothetical result, such as their actual risk of contracting the virus based on their behaviour and likely exposure. Only 7% of the sample responded that their risk would be higher on the primary outcome measure. Reasons for this are unclear.

While we found no evidence for a direct effect on protective behaviours of the terms used to describe antibody tests results, there was indirect evidence that perceiving no risk of future infection might reduce frequency of handwashing. This finding is tentative, given it is based on behavioural intentions in response to a hypothetical antibody-positive result. Nonetheless the potential for antibody testing to increase viral transmission must be considered alongside the potential benefits the tests might have in allowing the easing of lockdown restrictions. Clear communication about the ongoing need for handwashing, in particular, will be essential and raising public awareness of the main mechanisms through which SARS-CoV-2 is transmitted – through air and surfaces – might help improve adherence. This, in addition to acknowledgment of the imperfect nature of the tests, will give the public a more accurate representation of the meaning and implications of an antibody test result and a better understanding of how to reduce the risk of transmission. Such communications need to emphasise that transmission can occur through contact regardless of antibody status. Such communications also need to be rigorously evaluated to ensure their effectiveness at communicating these points both to those undergoing antibody tests as well as to general populations that are now having to learn to live with SARS-CoV-2.

It was notable that 85% of respondents said that they would probably or definitely have an antibody test if they were offered it. This suggests high interest but should be treated with caution not least because fewer undergo than express interest in undergoing tests (21).

Strengths and Limitations

This study provides the first experimental evidence for the potentially adverse impact on risk perceptions and protective behaviours of commonly used terms to describe SARS-CoV-2 antibody tests and their results. As such, it provides timely evidence to inform policy and research to mitigate these effects to realise the potential benefits of such tests.

For peer review only - http://bmjopen.bmj.com/site/about/guidelines.xhtml

The study has several limitations. First, participants were responding to a hypothetical test and asked to imagine that they had received a test result that had detected antibodies. The extent to which people responded using the information provided in the study is unknown. Some will likely have used other information about SARS-CoV-2 and their own experiences to answer the questions about risk. This is a limitation of all hypothetical scenario-based studies of this type but findings from such studies can generalise to clinical settings (22,23) although some caution is warranted. As antibody testing is not yet routinely being carried out in the UK, the impact of the wording used in the current study amongst those undergoing testing cannot be assessed. All scenarios used the term 'positive' so we were unable to examine any possible impact of the use of this term. In addition, due to the rapidity with which the current study was conducted, piloting of the materials in general population samples was not carried out. It is therefore possible that some of the information provided may have been misinterpreted.

Second, the protective behaviours of handwashing and physical distancing were measured using single items assessing behavioural intentions following a hypothetical test result.

Third, the sample size was insufficient to detect effect sizes that could be important at a population level. It is possible, for example, that the use of the terms Certificate or Passport might impact on risk perception, but the current study lacked the power to detect this.

Fourth, while quotas were used to achieve a sample broadly representative of the UK population, research panels are not representative of the general population (24,25). We found no evidence that the impact of the interventions in this study was modified by demographic characteristics of the participants, providing some reassurance about the generalisability of results across age groups, gender, educational level and geographical region of the UK.

Implications for research and policy

The results of this study have several implications for research and policy. Our finding that 85% of people were positively inclined towards having a test is encouraging for any future antibody testing programme. The effectiveness of antibody tests for SARS-CoV-2 will depend not only on high uptake, the extent and duration of any immunity conferred and the performance of a test, but also upon a good understanding of the meaning of tests results among those who participate. First, the use of the term Immunity should be avoided in phrases to describe antibody tests, whether described as Passports, Certificates or Tests. This has implications for the presentation of antibody testing by policy-makers and the media, as well as those considering the wording of test result letters sent to tested individuals. Second, research is needed to evaluate different ways of informing those

For peer review only - http://bmjopen.bmj.com/site/about/guidelines.xhtml

offered tests and receiving tests results to minimise the proportion erroneously perceiving an antibody-positive test result to mean no risk of becoming infected with the virus. It should also focus on maximising understanding that – regardless of antibody-status – anyone can indirectly transmit the virus by touching a contaminated surface and infecting the next surface they touch. Hand washing or sanitising therefore need to remain frequent. Research is also needed with those undergoing actual tests, powered to detect effects judged meaningful in the context of a population-based testing programme and involving measures of actual behaviour.

Conclusion

Interest in SARS-CoV-2 antibody testing is high – across many countries, employers and populations. While such testing could contribute to wider strategies to ease lock-down restrictions, their use may have an adverse impact on transmission-related behaviour. This appears to vary with the way the tests are described. Using the term Immunity (vs Antibody) to describe antibody tests increases the proportion of people believing that an antibody-positive result means they have no future risk of coronavirus, a perception that may be associated with less frequent handwashing and hence increased risk of transmission.

For peer review only

Figure 1 **Proportion believing an antibody-positive test result means 'no risk' of future infection** *Error bars show 95% confidence intervals*

See separate file for Figure 1

Figure 2 **Perceived meaning of an antibody-positive test result for future risk and intentions to reduce frequency of hand washing** *Error bars show 95% confidence intervals*

See separate file for Figure 2

For peer review only

For peer review only - http://bmjopen.bmj.com/site/about/guidelines.xhtml

WALLER et al FINAL BMJ Open R2 MS 20 July 2020

References

1. Hale T, Webster S, Petherick A, Phillips T, Kira B. *Oxford COVID-19 Government Response Tracker*, Blavatnik School of Government [Internet]. 2020. Available from: https://www.bsg.ox.ac.uk/research/research-projects/coronavirus-government-response-tracker

2. World Health Organization. *Critical preparedness, readiness and response actions for COVID-19* [Internet]. World Health Organization; 2020. (Interim Guidance 22 March 2020). Report No.: WHO/2019-nCoV/Community_Actions/2020.3. Available from: https://www.who.int/publications-detail/critical-preparedness-readiness-and-response-actions-for-covid-19

3. Studdert DM, Hall MA. Disease Control, Civil Liberties, and Mass Testing — Calibrating Restrictions during the Covid-19 Pandemic. *N Engl J Med* [Internet]. 2020 Apr 9; Available from: https://doi.org/10.1056/NEJMp2007637

4. Proctor K, Sample I, Oltermann P. 'Immunity passports' could speed up return to work after Covid-19. *The Guardian* [Internet]. 2020 Mar 30; Available from: https://www.theguardian.com/world/2020/mar/30/immunity-passports-could-speed-up-return-to-work-after-covid-19

5. Slotnick D. Delta's CEO said he would support an 'immunity passport' program or other steps to jumpstart travel as the airline reports its first quarterly loss in more than 5 years. *Business Insider* [Internet]. Available from: https://www.businessinsider.com/delta-first-quarter-coronavirus-air-travel-outlook-after-the-pandemic-2020-4

6. Merrick R. People could be given coronavirus 'immunity certificates' to leave lockdown early. *The Independent* [Internet]. 2020 Apr 2; Available from: https://www.independent.co.uk/news/uk/politics/coronavirus-uk-immunity-certificate-boris-johnson-test-germany-a9442211.html

7. Giugliano F. Mass Coronavirus Antibody Tests Have Serious Limits. *Bloomberg.com* [Internet]. 2020 Apr 24; Available from: https://www.bloomberg.com/opinion/articles/2020-04-24/coronavirus-mass-covid-19-antibody-tests-have-serious-limits

8. Forgey Q. Fauci: Coronavirus immunity cards for Americans are 'being discussed'. *POLITICO* [Internet]. Available from: https://www.politico.com/news/2020/04/10/fauci-coronavirus-immunity-cards-for-americans-are-being-discussed-178784

9. Sherwood D. Chile to push ahead with coronavirus 'release certificates' despite WHO warning. *Reuters* [Internet]. 2020 Apr 26; Available from: https://www.reuters.com/article/us-health-coronavirus-chile-idUSKCN2280NW

10. Adams ER, Anand R, Andersson MI, Auckland K, Baillie JK, Barnes E, et al. Evaluation of antibody testing for SARS-Cov-2 using ELISA and lateral flow immunoassays. *MedRxiv* Prepr. 2020 Jan 1;2020.04.15.20066407.

For peer review only - http://bmjopen.bmj.com/site/about/guidelines.xhtml

11. Altmann DM, Douek DC, Boyton RJ. What policy makers need to know about COVID-19 protective immunity. *The Lancet* [Internet]. 2020 Apr 27. Available from: https://www.thelancet.com/journals/lancet/article/PIIS0140-6736(20)30985-5/abstract

12. Loong T-W. Understanding sensitivity and specificity with the right side of the brain. *BMJ*. 2003;327(7417):716–719.

13. Marteau T, Saidi G, Goodburn S, Lawton J, Michie S, Bobrow M. Numbers or words? A randomized controlled trial of presenting screen negative results to pregnant women. Published in Affiliation with the International Society for Prenatal Diagnosis. *Prenat Diagn* 2000209714-718.

14. Marteau TM, Senior V, Sasieni P. Women's understanding of a "normal smear test result": experimental questionnaire based study. *BMJ*. 2001;322(7285):526–528.

15. World Health Organization. *'Immunity passports' in the context of COVID-19* [Internet]. World Health Organization. Report No.: WHO/2019-nCoV/Sci_Brief/Immunity_passport/2020.1. Available from: https://www.who.int/publications-detail/immunity-passports-in-the-context-of-covid-19

16. Jaki T, Vasileiou D. Factorial versus multi-arm multi-stage designs for clinical trials with multiple treatments. *Stat Med*. 2017 Feb 20;36(4):563–80.

17. Lohm D, Davis M, Flowers P, Stephenson N. 'Fuzzy' virus: indeterminate influenza biology, diagnosis and surveillance in the risk ontologies of the general public in time of pandemics. *Health Risk Soc*. 2015 Feb 17;17(2):115–31.

18. Paul, Slovic. "The Perception of Risk." Scientists Making a Difference: One Hundred Eminent Behavioral and Brain Scientists Talk about Their Most Important Contributions. Cambridge UP, 2016. 179-82. Web.

19. Fischhoff B, Brewer NT, Downs J. Communicating Risks and Benefits: An Evidence-Based User's Guide (FDA, 2011).

20. de Bruin WB, Fischhoff B, Millstein SG, Halpern-Felsher BL. Verbal and Numerical Expressions of Probability: 'It's a Fifty-Fifty Chance'. *Organ Behav Hum Decis Process*. 2000 Jan;81(1):115–31.

21. Cooke R, French DP. How well do the theory of reasoned action and theory of planned behaviour predict intentions and attendance at screening programmes? A meta-analysis. *Psychol Health*. 2008 Oct 1;23(7):745–65.

22. Wright AJ, Takeichi C, Whitwell SCL, Hankins M, Marteau TM. The impact of genetic testing for Crohn's disease, risk magnitude and graphical format on motivation to stop smoking: an experimental analogue study. *Clin Genet*. 2008 Apr;73(4):306–14.

23. Hollands GJ, Whitwell SCL, Parker RA, Prescott NJ, Forbes A, Sanderson J, et al. Effect of communicating DNA based risk assessments for Crohn's disease on smoking cessation: randomised controlled trial. *BMJ* [Internet]. 2012 Jul 20;345. Available from: https://www.bmj.com/content/345/bmj.e4708

For peer review only - http://bmjopen.bmj.com/site/about/guidelines.xhtml

24. ONS. Internet users, UK - Office for National Statistics [Internet]. Office for National Statistics; 2019 May. Available from: https://www.ons.gov.uk/businessindustryandtrade/itandinternetindustry/bulletins/internetusers/2019

25. Wright KB. Researching Internet-Based Populations: Advantages and Disadvantages of Online Survey Research, Online Questionnaire Authoring Software Packages, and Web Survey Services. *J Comput-Mediat Commun*. 2005;10(3).

Funding

Data collection for this study was funded by a block UK government grant to the Behavioural Insights Team. JW is funded by a career development fellowship from Cancer Research UK (ref C7492/A17219). GJR is funded by the National Institute for Health Research Health Protection Research Unit (NIHR HPRU) in Emergency Preparedness and Response at King's College London in partnership with Public Health England (PHE), in collaboration with the University of East Anglia. The views expressed in this paper are those of the authors and not necessarily those of UK government, Cancer Research UK, NIHR or Public Health England.

Competing interests

All authors have completed the Unified Competing Interest form (available on request from the corresponding author) and declare: no support from any organisation for the submitted work; and no financial relationships with any organisations that might have an interest in the submitted work in the previous three years. HWWP declares consultancy fees from Babylon Health; all authors declare no other relationships or activities that could appear to have influenced the submitted work.

Transparency declaration

The authors affirm that the manuscript is an honest, accurate, and transparent account of the study being reported; that no important aspects of the study have been omitted; and that any discrepancies from the study as originally planned have been explained.

For peer review only

Data sharing statement

Anonymised data will be made available upon reasonable request.

Author contribution statement

The study was conceptualised by TMM, JW & GJR. AM completed data collection. JW & HP analysed the data. All authors contributed to, and approved, the final manuscript.

Acknowledgements

We thank Steve Reicher for comments on an earlier draft of the study protocol.

For peer review only

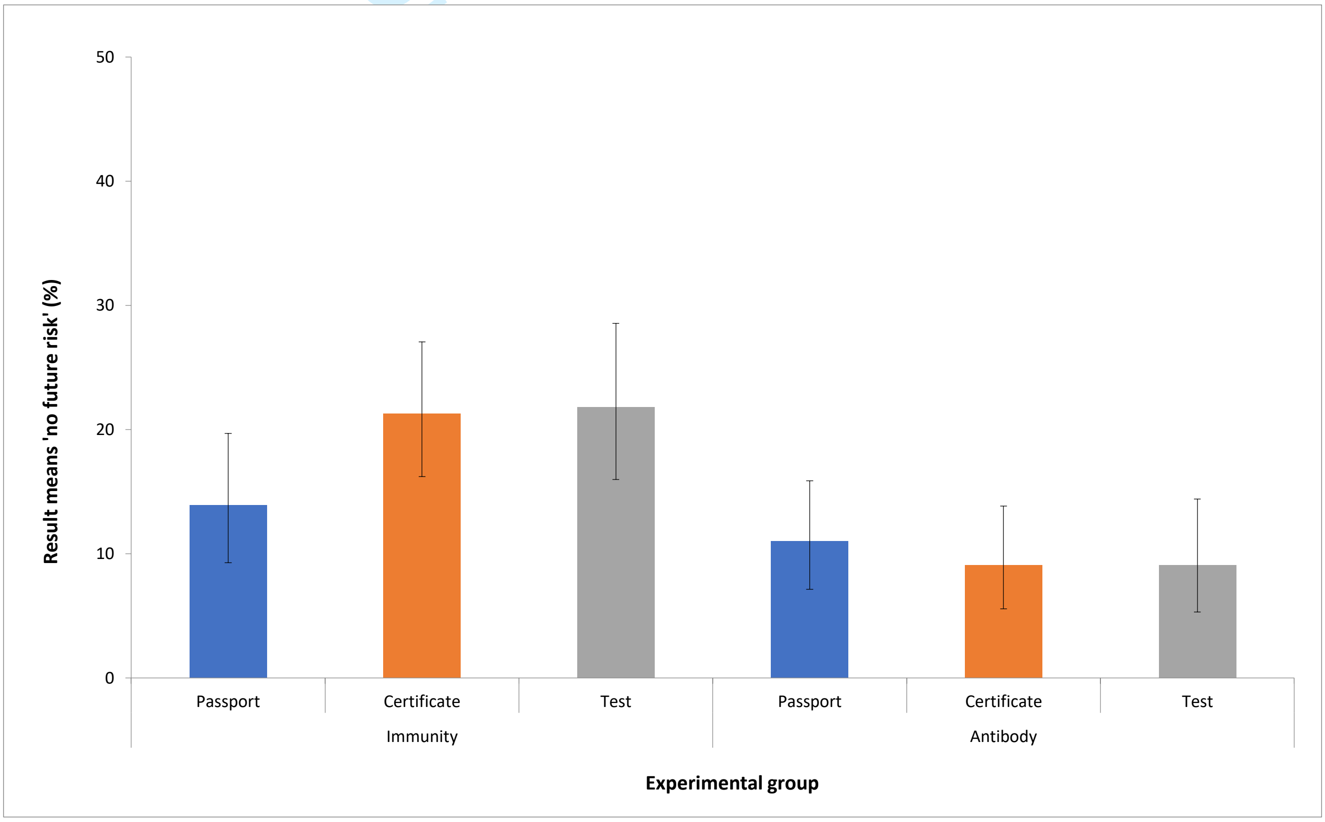

Result means 'no future risk' (%)
50
40
30
20
10
0
Passport
Certificate
Test
Passport
Certificate
Test
Immunity
Antibody
Experimental group

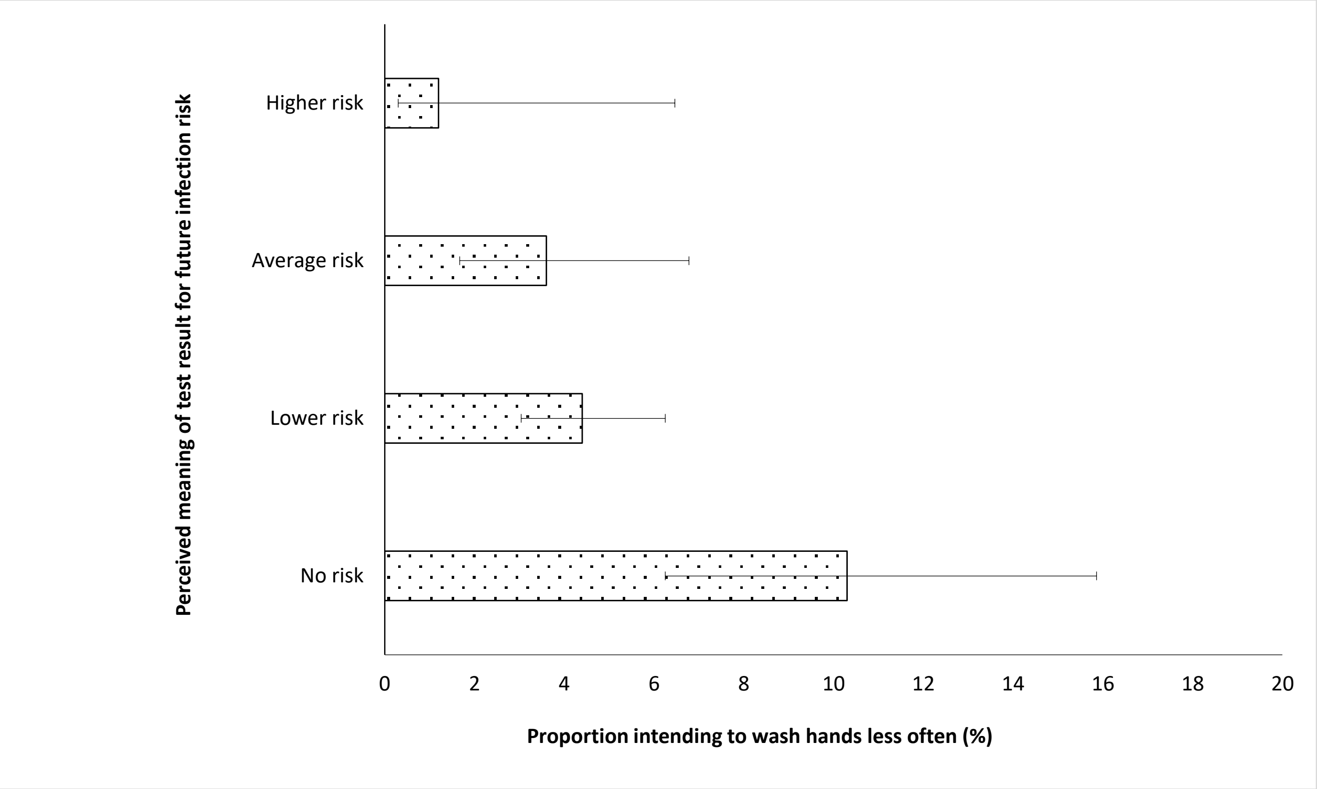
Perceived meaning of test result for future infection risk
Higher risk
Average risk
Lower risk
No risk
0 2 4 6 8 10 12 14 16 18 20
Proportion intending to wash hands less often (%)

Supplementary Materials

S1 Interventions

Intervention A

Immunity Passport

Scientists are developing tests to see who has already had coronavirus.

No test is 100% effective.

This means that those who test 'positive' would have:

- Lower risk of catching coronavirus in the future - and therefore also
- Lower risk of passing it on to others

Those who test 'positive' would get an immunity passport.

They could return to work early.

Intervention B

Immunity Certificate

Scientists are developing tests to see who has already had coronavirus.

No test is 100% effective.

This means that those who test 'positive' would have:

- Lower risk of catching coronavirus in the future - and therefore also
- Lower risk of passing it on to others

Those who test 'positive' would get an immunity certificate.

They could return to work early.

Intervention C

Immunity Test

Scientists are developing tests to see who has already had coronavirus.

No test is 100% effective.

This means that those who test 'positive' would have:

- Lower risk of catching coronavirus in the future - and therefore also
- Lower risk of passing it on to others

Those who test 'positive' would get a result showing immunity.

They could return to work early.

Intervention D

Antibody Passport

Scientists are developing tests to see who has already had coronavirus.

No test is 100% effective.

This means that those who test 'positive' would have:

- Lower risk of catching coronavirus in the future - and therefore also
- Lower risk of passing it on to others

Those who test 'positive' would get an antibody passport.

They could return to work early.

Intervention E

Antibody Certificate

Scientists are developing tests to see who has already had coronavirus.

No test is 100% effective.

This means that those who test 'positive' would have:

- Lower risk of catching coronavirus in the future - and therefore also
- Lower risk of passing it on to others

Those who test 'positive' would get an antibody certificate.

They could return to work early.

Intervention F

Antibody Test

Scientists are developing tests to see who has already had coronavirus.

No test is 100% effective.

This means that those who test ‘positive’ would have:

- Lower risk of catching coronavirus in the future - and therefore also
- Lower risk of passing it on to others

Those who test ‘positive’ would get a result showing antibodies.

They could return to work early.

For peer review only

S2. Measures

Primary outcome

I Imagine you were given *[an immunity passport/an immunity certificate/a result showing immunity/an antibody passport/an antibody certificate/a result showing antibodies]*

which one of the following statements is true*: [order of response options to be randomised]*

1. I have no risk of catching coronavirus in the future
2. I have a lower risk of catching coronavirus in the future
3. I have an average risk of catching coronavirus in the future
4. I have a higher risk of catching coronavirus in the future

Secondary outcomes

II Imagine you were given *[an immunity passport/an immunity certificate/a result showing immunity/an antibody passport/an antibody certificate/a result showing antibodies]*

how likely is it that you will get coronavirus at some point in the future?

Please answer on a scale from 0 % to 100% where 0% means no chance and 100% means certain.

0% --100%

III Imagine that you were given *[an immunity passport/an immunity certificate/a result showing immunity/an antibody passport/an antibody certificate/a result showing antibodies]*

would you wash your hands with soap and water or sanitiser:

1. Much more than now
2. More than now
3. Same as now
4. Less than now
5. Much less than now

IV Imagine you were given *[an immunity passport/an immunity certificate/a result showing immunity/an antibody passport/an antibody certificate/a result showing antibodies]*

would you avoid physical contact with others outside of your home:

1. Much more than now
2. More than now
3. Same as now
4. Less than now
5. Much less than now

V If you were offered a test today by the NHS to check whether you have ever had coronavirus, would you have it?

1. Yes, definitely
2. Yes, probably
3. No, probably not
4. No, definitely not

For peer review only

Supplementary Table 1 – Logistic regression analysis examining impact of Test type and Result type on protective behaviours

	Intend to wash hands less			Intend to avoid contact less		
	% responding less/much less in each sub-group % (95% CI)	Mutually adjusted (n=1204)	Adjusted for demographics[1] (n=1179)	% responding less/much less in each sub-group % (95% CI)	Mutually adjusted (n=1204)	Adjusted for demographics[1] (n=1179)
Test Type						
Antibody (n=603)	4.1 (2.7-6.1)	Ref	Ref	17.2 (104)	Ref	Ref
Immunity (n=601)	5.7 (4.0-7.8)	1.38 (0.43-4.45)	1.62 (0.46-5.73)	22.1 (133)	1.40 (0.82-2.39)	1.46 (0.85-2.50)
Result Type						
Test (n=354)	3.4 (1.8-5.9)	Ref	Ref	19.2 (68)	Ref	Ref
Certificate (n=444)	6.8 (4.6-9.5)	2.07 (0.72-6.00)	2.47 (0.77-7.96)	21.6 (96)	1.16 (0.68-1.96)	1.14 (0.67-1.96)
Passport (n=406)	4.2 (2.5-6.6)	1.29 (0.41-4.01)	1.79 (0.52-6.16)	18.0 (73)	0.99 (0.58-1.69)	1.02 (0.59-1.76)
Test by Result interaction						
Certificate by Immunity result		0.98 (0.25-3.95)	0.80 (0.18-3.56)		1.00 (0.49-2.01)	0.94 (0.46-1.93)
Passport by Immunity result		0.96 (0.21-4.40)	0.58 (0.11-2.98)		0.89 (0.43-1.87)	0.84 (0.40-1.78)

[1]Fully adjusted model includes age (with quadratic term), gender, education level and region (coded as per Table 1)

For peer review only

Supplementary Table 2 – Logistic regression examining impact of Test and Result type on intention to have the test

	Do not intend to have the test		
	Proportion who would not have test in each sub-group % (95% CI)	Mutually adjusted (n=1204)	Adjusted for demographics[1] (n=1179)
Test Type			
Antibody (n=603)	13.1 (10.5-16.1)	Ref	Ref
Immunity (n=601)	16.5 (13.6-19.7)	1.02 (0.57-1.81)	0.97 (0.54-1.74)
Result Type			
Test (n=354)	15.5 (11.9-19.7)	Ref	Ref
Certificate (n=444)	14.9 (11.7-18.5)	0.71 (0.39-1.28)	0.63 (0.34-1.15)
Passport (n=406)	14.0 (10.8-17.8)	0.80 (0.54-1.42)	0.82 (0.46-1.47)
Test by Result interaction			
Certificate by Immunity result		1.65 (0.75-3.63)	1.91 (0.84-4.31)
Passport by Immunity result		1.23 (0.55-2.75)	1.22 (0.54-2.78)

[1]Fully adjusted model includes age (with quadratic term), gender, education level and region (coded as per Table 1)

For peer review only

For peer review only - http://bmjopen.bmj.com/site/about/guidelines.xhtml

Supplementary Table 3 – Logistic regression analysis examining impact of perceived risk on anticipated behaviour

	Intend to wash hands less			Intend to avoid contact less		
	% responding less/much less in each sub-group % (95% CI)	Unadjusted (n=1204)	Adjusted for demographics[1] (n=1179)	% responding less/much less in each sub-group % (95% CI)	Unadjusted (n=1204)	Adjusted for demographics[1] (n=1179)
Perceived meaning of result						
Some residual risk (n=1030)	4.0 (2.8-5.3)	Ref	Ref	18.8 (16.5-21.4)	Ref	Ref
No residual risk of future infection (n=175)	10.3 (6.3-15.9)	**2.78 (1.56-4.97)**	**2.32 (1.25-4.28)**	24.7 (18.5-31.8)	1.41 (0.97-2.06)	1.37 (0.93-2.03)

[1]Fully adjusted model includes age (with quadratic term), gender, education level and region (coded as per Table 1)

For peer review only
